# Active targeting towards bone cancer and metastases by pamidronate-bridged dinuclear platinum(II) complexes

**DOI:** 10.3389/fchem.2026.1945537

**Published:** 2026-09-22

**Authors:** Anna Maria Di Cosola, Alessandra Barbanente, Luisa D’Anna, Simona Rubino, Alessio Terenzi, Mauro Niso, Nicola Margiotta

**Affiliations:** 1 Dipartimento di Chimica, Università degli Studi di Bari Aldo Moro, Bari, Italy; 2 Dipartimento di Scienze e Tecnologie Biologiche Chimiche e Farmaceutiche, Università degli Studi di Palermo, Palermo, Italy; 3 Dipartimento di Farmacia-Scienze del Farmaco, Università degli Studi di Bari Aldo Moro, Bari, Italy

**Keywords:** bone cancer, bisphosphonates, bone metastases, DNA g-quadruplex, kiteplatin, oxaliplatin derivatives, pamidronate, platinum(II) complexes

## Abstract

Bisphosphonates (BPs) are clinically established bone-seeking agents that strongly bind to hydroxyapatite and selectively accumulate at sites of active bone remodeling. Among nitrogen-containing BPs, pamidronate (PAM) is widely used for the management of cancer-related bone complications and has also been associated with antiproliferative and pro-apoptotic effects in several tumor models. These properties make PAM an attractive ligand for the development of potential bone-targeted anticancer agents, particularly in the context of primary bone tumors and bone metastases, which are difficult to treat and are frequently associated with severe pain and skeletal complications. Platinum-based chemotherapeutics are effective anticancer agents, but their clinical use is often limited by poor tissue selectivity, systemic toxicity, and drug resistance. In this work, we report the synthesis and full characterization of two novel dinuclear platinum(II)–PAM complexes in which PAM acts as a bridging ligand between two platinum centers containing either an oxaliplatin-derived [Pt(1*R*,2*R*-diaminocyclohexane)]^2+^ unit or a kiteplatin-derived [Pt(*cis*-1,4-diaminocyclohexane)]^2+^ unit. The new compounds were characterized by multinuclear NMR spectroscopy and ESI-MS, and their chemical stability was investigated under physiological and mildly acidic conditions. Their reactivity toward DNA models and their *in vitro* antiproliferative activity against breast adenocarcinoma and osteosarcoma cells were also evaluated. Overall, these results highlight the influence of both the bisphosphonate scaffold and the diamine carrier ligand on the chemical and biological properties of dinuclear platinum(II) complexes, supporting their further investigation as potential agents for the treatment of bone malignancies.

## 1 Introduction

Nitrogen-containing bisphosphonates (BPs) represent a well-established class of bone-seeking agents widely used in the clinical management of osteoporosis, Paget’s disease, malignancy-associated hypercalcemia, and osteolytic bone metastases (Conry et al., 2016). Their therapeutic relevance is mainly related to their strong affinity for hydroxyapatite (HA), the principal inorganic component of bone, which enables their accumulation at sites of active bone remodeling. Once localized at the bone surface, these compounds inhibit osteoclast-mediated bone resorption, reduce skeletal-related events (SREs), improve bone integrity, and alleviate tumor-associated bone pain. Among clinically used bisphosphonates, zoledronate (ZL) and pamidronate (PAM) have attracted particular interest in the oncology field. Beyond their classical antiresorptive activity, increasing evidence indicates that nitrogen-containing BPs may also exert direct and indirect antitumor effects. These effects include inhibition of tumor cell adhesion and invasion, suppression of angiogenesis, modulation of immune responses, interference with matrix metalloproteinases, and induction of apoptosis (Wu et al., 2020). ZL has been extensively investigated as an adjuvant agent in bone cancer and bone metastases, showing the ability to impair tumor cell adhesion, proliferation, invasion, and survival in several cancer models, including leukemia (Kimura et al., 2004) and breast cancer (Senaratne and Colston, 2001). In osteosarcoma models, ZL has been reported to impair tumor cell proliferation, survival, and vasculogenic mimicry (Chang et al., 2012; Fu et al., 2011). Additional studies demonstrated its ability to potentiate antitumor therapies (Moriceau et al., 2010), suppress osteosarcoma-induced bone lesions (Labrinidis et al., 2009), and inhibit metastatic progression *in vivo* (Koto et al., 2009). Similarly, PAM has been reported to display antiproliferative and pro-apoptotic activity in several tumor cell lines, particularly in breast cancer models, and to interfere with tumor cell invasiveness and survival pathways (Wada et al., 2006). For these reasons, the biological and pharmacological properties of BPs make them particularly relevant in the context of bone tumors and skeletal metastases. Primary bone tumors, including osteosarcoma, chondrosarcoma, and Ewing’s sarcoma, are relatively rare but often aggressive malignancies. More frequently, bone lesions arise as secondary metastases from primary tumors such as breast, prostate, lung, kidney, and thyroid cancers (Buenrostro et al., 2016). Bone metastases are common in advanced-stage disease, with a high incidence in patients with breast and prostate cancer (Gnant et al., 2012; Saad and Lattouf, 2012). Their development is driven by complex interactions between cancer cells and the bone microenvironment, involving osteoblasts, osteoclasts, fibroblasts, and immune cells (Criscitiello et al., 2015; Giles et al., 2016). This bidirectional crosstalk disrupts normal bone remodeling and promotes a pathological cycle that leads to skeletal fragility, pain, fractures, reduced mobility, and poor quality of life (Coleman et al., 2020). Platinum-based chemotherapeutic agents, including cisplatin, carboplatin, and oxaliplatin, remain central components of anticancer therapy due to their broad cytotoxic activity. Their mechanism of action is mainly associated with intracellular activation followed by the formation of covalent DNA adducts, which interfere with DNA replication and transcription and ultimately lead to cell death (Krzeszinski and Wan, 2015). However, the clinical efficacy of platinum drugs is limited by poor tissue selectivity, acquired or intrinsic resistance, and severe off-target toxicities, including nephrotoxicity, neurotoxicity, and ototoxicity (Elmorsy et al., 2024). These limitations have stimulated the development of drug targeting and delivery (DTD) strategies aimed at improving the selective accumulation of cytotoxic agents at pathological sites while reducing systemic exposure. Active DTD approaches, in particular, rely on carrier ligands capable of directing therapeutic agents toward specific tissues or cells (Russell, 2011). Consequently, targeted delivery systems have attracted considerable interest as a strategy to enhance therapeutic efficacy and reduce unwanted toxicity (Iafisco et al., 2008; Torres Martin de Rosales et al., 2009). In bone-targeted therapy, the conjugation of platinum-based cytotoxic fragments to bone-seeking ligands, such as pyrophosphate and BPs, represents a promising approach. By exploiting the affinity of BPs for bone mineral surfaces, platinum–BP conjugates may promote preferential drug accumulation in bone tumors and metastases while maintaining the cytotoxic potential of the platinum moiety. Several platinum(II) complexes containing phosphonate or bisphosphonate ligands have been investigated as potential bone-targeted anticancer agents (Nadar et al., 2021; Piccinonna et al., 2012). An early study by Galanski et al. demonstrated promising *in vitro* antitumor activity for osteotropic platinum(II) complexes incorporating phosphonate ligands (Galanski et al., 2003). These findings stimulated the development of platinum(II)–BP conjugates designed to improve drug accumulation in bone metastases and enhance antitumor efficacy (Nakatake et al., 2011; Xue et al., 2010). More recently, radioactive platinum–bisphosphonate derivatives demonstrated selective targeting of bone tissues with high metabolic activity, further supporting the potential of this approach (Nadar et al., 2020). Based on these findings, Margiotta et al. synthesized dinuclear platinum-bisphosphonate (Pt-BP) complexes in which 2-ammonium-1-hyroxyethane-1,1-diyl bisphosphonate (AHBP) was employed for bridging two platinum residues, either *cis*-[Pt(NH_3_)_2_]^2+^ (related to cisplatin, *cis*-[PtCl_2_(NH_3_)_2_]) or [Pt(*cis*-1,4-DACH)]^2+^ (related to kiteplatin [PtCl_2_(*cis*-1,4-DACH)]; DACH = diaminocyclohexane) (Margiotta et al., 2012). Complexes with the kiteplatin moiety were found to be more active, likely due to stronger DNA interactions. These dinuclear [{Pt(*cis*-1,4-DACH)}_2_(BP)]^+^ compounds were also able to overcome cisplatin and multidrug resistance in colorectal cancer cells. Additionally, they also demonstrated that radioactive ^195m^Pt-BP complexes showed high bone affinity and targeted bone metastases more effectively than ^195m^Pt–cisplatin (Nadar et al., 2020). Notably, platinum–bisphosphonate conjugates incorporating the *cis*-1,4-DACH ligand, an isomeric form of the oxaliplatin diamine ligand 1*R*,2*R*-DACH, exhibit promising activity against cisplatin-resistant tumor models (Gandin et al., 2014; Gibson, 2016). Building on this background, we recently reported two dinuclear platinum(II) complexes in which ZL acts as a bridging ligand between the two platinum moieties derived from oxaliplatin and from kiteplatin (Barbanente et al., 2025b). The oxaliplatin-based ZL complex showed higher cytotoxicity than the kiteplatin-based analogue, particularly against HepG2 and MCF-7 cells. Moreover, conjugation of the [Pt(1*R*,2*R*-DACH)]^2+^ and [Pt(*cis*-1,4-DACH)]^2+^ units with ZL improved the anticancer activity of both free ZL and oxaliplatin in MCF-7wt, HepG2, and PANC-1 cancer cell lines, while comparable activity was observed in HEK-293 cells. These findings encouraged us to extend this investigation to PAM, and to evaluate whether the use of a different BP scaffold could modulate the chemical and biological behavior of this class of dinuclear Pt-BP compounds. PAM is a clinically approved nitrogen-containing bisphosphonate with established bone affinity and reported antitumor properties, making it an attractive bridging ligand for the development of potential bone-targeted platinum (II) complexes. In the present work, we report the synthesis and characterization of two new dinuclear platinum (II)–PAM complexes [{Pt(1*R*,2*R*-DACH)}_2_(PAM)]^+^ (**PtBP3**) and [{Pt(*cis*-1,4-DACH)}_2_(PAM)]^+^ (**PtBP4**), and compare their biological behavior with that of the previously described ZL analogues [{Pt(1*R*,2*R*-DACH)}_2_(ZL)]^+^ (**PtBP1**) and [{Pt(*cis*-1,4-DACH)}_2_(ZL)]^+^ (**PtBP2**) (Barbanente et al., 2025b). In this study, **PtBP1** and **PtBP2** were also evaluated for the first time in U2OS (osteosarcoma) cells, allowing a direct comparison with the newly synthesized PAM-containing complexes. The new compounds were characterized by multinuclear NMR spectroscopy and ESI-MS, and their chemical stability was assessed under physiological and mildly acidic conditions, simulating the tumor microenvironment. Their antiproliferative activity was investigated in MCF7wt breast cancer cells, frequently associated with bone metastases, and in U2OS osteosarcoma cells, representative of primary bone malignancies. In addition to standard MTT assays, the effects of the complexes on cellular DNA content were evaluated, and the possible involvement of oxidative stress was explored by assessing cell viability in the presence of the antioxidant α-tocopherol. Overall, this study aims to clarify the influence of the bisphosphonate scaffold on the behavior of dinuclear platinum complexes and to contribute to the design of platinum(II)-based therapeutics with potential improved selectivity toward bone malignancies.

## 2 Experimental section

### 2.1 Starting materials and instrument details

High purity commercial reagents and solvents were used without further purification. ESI-MS spectra were obtained with an electrospray interface and a quadrupole time-of-flight mass spectrometer (Agilent 6530 Series Accurate-Mass Quadrupole Time-of-Flight (Q-TOF) LC-MS; Agilent Technologies Italia S.p.A.; Cernusco sul Naviglio, Milano, Italy). 1D ^1^H-, ^31^P-, and ^195^Pt-NMR and 2D [^1^H–^13^C]-HSQC and HMBC spectra were recorded on Bruker Avance DPX 300 MHz (Bruker Italia S.r.L., Milano, Italy) instrument. Standard pulse sequences were used for ^1^H, ^31^P{^1^H}, and ^195^Pt{^1^H} (300, 121.5, and 64.5 MHz, respectively) 1D spectra. ^1^H chemical shifts were referenced using the internal residual peak of the solvent (D_2_O: 4.80 ppm) or 3-trimethylsilylpropionic acid (TSP; 0 ppm), ^31^P chemical shifts were referenced to external H_3_PO_4_ (85% w/w; 0 ppm), while the ^195^Pt chemical shifts were referenced to external K_2_[PtCl_4_] in D_2_O fixed at −1628 ppm. Elemental analyses were performed with an Eurovector EA 3000 CHN instrument (Eurovector S.p.A., Milano, Italy). For pH measurements, a Crison Micro-pHmeter model 2002 was used, equipped with micro-combined electrodes (5- and 3 mm diameter) and calibrated with Crison standard buffer solutions at pH 4.01, 7.02 and 10.00. pH readings taken on solutions in D_2_O are indicated with pH* and are not corrected considering the effect of deuterium on glass electrodes (Feltham and Hayter, 1964).

### 2.2 Synthesis of pamidronate

3-ammonium-1-hydroxypropane-1,1-diyl-bisphosphonic acid (pamidronic acid, PAM), was prepared according to an already reported procedure (Guénin et al., 2007). The elemental analysis and the spectroscopic features of PAM were consistent with data reported in the literature.

### 2.3 Synthesis of the platinum precursors

[PtI_2_(1*R*,2*R*-DACH)] (DACH = diaminocyclohexane) (Dhara, 1970) [Pt(OSO_3_)(OH_2_)(1*R*,2*R*-DACH)] [PtCl_2_(*cis*-1,4-DACH)] (kiteplatin) [Pt(OSO_3_)(OH_2_)(*cis*-1,4-DACH)] [{Pt(1*R*,2*R*-DACH)}_2_(ZL)]NO_3_ (**PtBP1**) and [{Pt(*cis*-1,4-DACH)}_2_(ZL)]NO_3_ (**PtBP2**) were prepared as already reported (Barbanente et al., 2025b; Dhara, 1970; Ranaldo et al., 2008). The elemental analyses and the spectroscopic features of the Pt precursor complexes were consistent with the data reported in the literature.

### 2.4 Preparation of [{Pt(1*R*,2*R*-DACH)}_2_(PAM)]NO_3_ (PtBP3)

Pamidronic acid (PAM) was dissolved in water (9.7 mL) at 60 °C and treated with a solution of [Pt(OSO_3_)(OH_2_)(1*R*,2*R*-DACH)] (40 mg, 0.09 mmol in 6.6 mL of H_2_O). The resulting solution was kept under magnetic stirring at 60 °C for 3 h, during which the pH was maintained at a constant value of approximately 4.0 by adding aliquots of KOH (1.0 M). Ba(OH)_2_·8H2O was then added (28.4 mg, 0.09 mmol) and the resulting suspension was stirred for 10 min at room temperature. The white solid (BaSO_4_) was removed by filtering the mother liquor through celite. The solution was then brought to pH approximately 2 by adding HNO_3_ (1.0 M) and concentrated to a volume of 2 mL. The concentrated solution was then treated with acetone to induce precipitation of the desired product as a white solid with NO_3_^−^ as counterion. The precipitate was left to stand at 4 °C for 30 min and then isolated by centrifugation, washed 3 times with acetone (every washing was followed by centrifugation) and dried under a flow of anhydrous air. Yield: 57.1% (19.8 mg). Elemental analysis, calculated for [{Pt(1*R*,2*R*-DACH)}_2_(PAM)]NO_3_‧3H_2_O (C_15_N_6_P_2_Pt_2_O_10_H_35_‧3H_2_O): C, 18.65; H, 4.28; N, 8.70%. Found: C, 18.23; H, 4.29; N, 8.30%. ESI-MS(+): calculated for [Pt(1*R*,2*R*-DACH)}_2_(PAM)]^+^ [C_15_H_35_N_5_O_7_P_2_Pt_2_+H]^+^, *m/z* = 850.1283; found [M + H]^+^, *m/z* = 850.0885. ^1^H NMR (D_2_O): 3.29 (2H, CH(1)), 2.21 (2H, CH(2)), 2.31 (1H, CH(4)), 2.53 (1H, CH(5)), 1.34 (2H, CH(6/9ax)), 2.09 (2H, CH(6/9eq)), 1.17 (2H, CH(7/8ax)), 1.62 (2H, CH(7/8eq) ppm. ^31^P{^1^H} NMR (D_2_O): 39.48, 38.98 ppm ^195^Pt{^1^H} NMR (D_2_O): 1668.71 ppm ^13^C{^1^H} NMR (D_2_O): 68 (C(4)), 65.61 (C(5)), 38.78 (C(1)), 34.68 (C(6/9)), 33.89 (C(2)), 26.93 (C(7/8)), 79.82 (C(3)) ppm. Numbering of atoms is reported in Figure 1.

**FIGURE 1:**
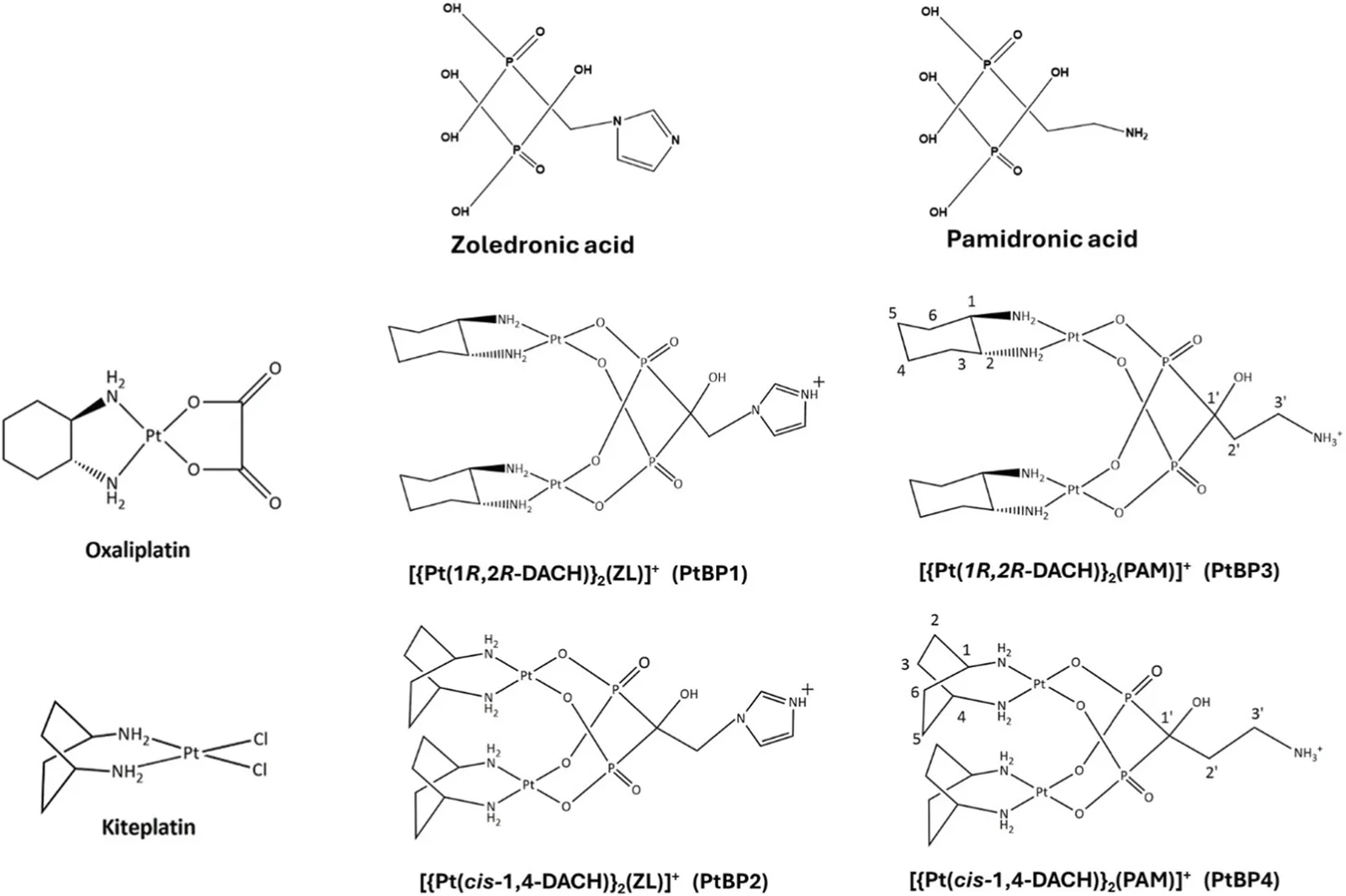
Structures of oxaliplatin, kiteplatin and their corresponding previously reported dinuclear zoledronate-bridged analogues **PtBP1** and **PtBP2** (Barbanente et al., 2025b). The new pamidronate-based complexes [{Pt(1*R*,2*R*-DACH)}_2_(PAM)]^+^ (**PtBP3**) and [{Pt(*cis*-1,4-DACH)}_2_(PAM)]^+^ (**PtBP4**), developed and investigated in this work, are also shown (DACH = diaminocyclohexane, ZL = zoledronic acid, PAM = pamidronic acid). Counter-anions are omitted.

### 2.5 Preparation of [{Pt(*cis*-1,4-DACH)}_2_(PAM)]NO_3_ (PtBP4)

PAM (10.1 mg, 0.043 mmol) was dissolved in water (9.7 mL) at 45 °C and treated with a solution of [Pt(OSO_3_)(OH_2_)(*cis*-1,4-DACH)] (40 mg, 0.094 mmol in 5.8 mL of H_2_O). The resulting solution was kept under stirring at 45 °C overnight. Ba(OH)_2_∙8H2O was then added (29.7 mg, 0.094 mmol) and the suspension was kept under stirring for 10 min at room temperature. The white solid obtained (BaSO_4_) was separated by filtration through celite. The obtained solution was then brought to pH approximately 2 by adding HNO_3_ (1.0 M) and concentrated to a volume of approximately 3.5 mL. Acetone was then added to induce the precipitation of the desired product as a white solid with NO_3_^−^ as counterion. The precipitate was left to stand at 4 °C for 30 min and then isolated by centrifugation, washed 3 times with acetone (every washing followed by centrifugation) and dried under a flow of anhydrous air. Yield: 69.6% (27.3 mg). Elemental analysis, calculated for [{Pt(*cis*-1,4-DACH)}_2_(PAM)]NO_3_ ‧ 4H_2_O (C_15_N_6_P_2_Pt_2_O_10_H_35_‧4H_2_O): C, 18.31; H, 4.40; N, 8.54%. Found: C, 17.90; H, 4.06; N, 8.79%. ESI-MS (+): calculated for [{Pt(*cis*-1,4-DACH)}_2_(PAM)]^+^ [C_15_H_35_N_5_ O_7_P_2_Pt_2_+H]^+^, *m/z* = 850.1283; found [M + H]^+^, *m/z* = 850.0787. ^1^H NMR (D_2_O): 4.96 (2H, NH_2_(a)), 5.31 (2H, NH_2_(a')), 3.31 (2H, CH(1)), 2.24 (2H, CH(3)), 3.14 (2H, CH(4/7)), 1.87 (8H, CH(5/6/8/9)) ppm. ^195^Pt{^1^H} NMR (D_2_O): 1596.57 ppm ^13^C{^1^H} NMR (D_2_O): 49.64 (C(4/7)), 38.92 (C(1)), 32.85 (C(2)), 24.78 (C(5/6/8/9)), 78.22 (C(3)) ppm. Numbering of atoms is reported in Figure 1.

### 2.6 Stability studies in physiological and weakly acidic environments

The stability of complexes **PtBP3** and **PtBP4** was evaluated by ^31^P{^1^H} NMR spectroscopy at 37 °C in buffer solutions. Each Pt complex (∼3 mg) was dissolved in 0.8 mL of D_2_O containing 50 mM 4-(2-hydroxyethyl)-1-piperazineethane sulfonic acid (HEPES buffer) (pH* = 7.4 or 6.5) and 120 mM NaCl. The resulting solutions were transferred to NMR tubes and kept at 37 °C. The ^31^P NMR spectra were recorded at defined times over a period of 7 days.

### 2.7 Reactivity studies with 5′-GMP in physiological environment

Each complex (2 mg, 0.002 mmol) was dissolved in 0.8 mL of D_2_O containing HEPES buffer 50 mM (pH* = 7.4) and 120 mM NaCl to simulate the physiological environment. After the addition of 4.5 mg (0.011 mmol) of guanosine 5′-monophosphate sodium salt (5′-GMP–Na·6H_2_O) the reaction mixtures were transferred to an NMR tube and kept at 37 °C. The reaction with 5′-GMP was monitored for a period of 7 days by recording ^1^H- and ^31^P{^1^H}-NMR spectra at defined time intervals.

### 2.8 Reactivity with 9-Ethylguanine and DNA oligonucleotides

9-Ethylguanine (9-EtG) was obtained from Sigma-Aldrich (Merck) and used without further purification. The oligonucleotide c-Myc (5’-TGA​GGG​TGG​GTA​GGG​TGG​GTA​A-3’ was obtained from Sigma-Aldrich (Merck) with desalted-grade purity in lyophilized form. The oligonucleotide solution was prepared by resuspending the lyophilized NH_4_^+^ containing buffer to favour the formation of the G-quadruplex secondary structure. To promote the folding of the G4 secondary structure, the solutions were heated to 90 °C for 5 min and then gradually cooled to room temperature overnight. Stock solutions of Pt complexes **PtBP3** and **PtBP4** were prepared in MilliQ water. Stock solutions of 9-EtG and c-Myc were prepared using ammonium acetate 100 mM. Final mixtures were prepared using ammonium acetate 100 mM with metal complexes at 10 μM concentration and DNA models at 1 μM concentration. High-Resolution Accurate Mass (HRAM) spectra were acquired using a Thermo Fisher Exploris Orbitrap 120 during the direct in-source infusion of standard solutions of 9-EtG or c-Myc mixed with **PtBP3** and **PtBP4** (after incubation at 37 °C for 24 h and 7 days). For the experiment with 9-EtG, Infusion was performed at a flow rate of 5 μL/min into a HESI source under the following conditions: positive ion mode with a spray voltage set to 2500 V, an ion transfer tube temperature of 280 °C, a sheath gas flow (arbitrary units) of 5, an auxiliary gas flow (arbitrary units) of 5, Data acquisition was carried out in the *m/z* range of 150–2000. For the experiment with c-Myc, infusion was performed at a flow rate of 5 μL/min into a HESI source under the following conditions: negative ion mode with a spray voltage set to −3000 V, an ion transfer tube temperature of 300 °C, a sheath gas flow (arbitrary units) of 10, an auxiliary gas flow (arbitrary units) of 0, Data acquisition was carried out in the *m/z* range of 150–3,000.

### 2.9 *In vitro* cytotoxicity studies

All Pt-BPs complexes were tested on MCF7wt and U2OS cell lines. For *in vitro* cytotoxicity studies MCF7wt cells were cultured in Dulbecco’s Modified Eagle’s Medium. This medium was supplemented with 10% (v/v) Fetal Bovine Serum, 1% (v/v) Glutamine and 1% (v/v) Penicillin–Streptomycin. Cells were cultivated at 37 °C with 5% CO_2_ at saturated humidity. U2OS cells were grown in McCoy’s 5a Medium Modified supplemented with 10% (v/v) Fetal Bovine Serum, 1% (v/v) Glutamine and 1% (v/v) Penicillin–Streptomycin. Cells were grown at 37 °C with 5% CO_2_ at saturated humidity. All cell culture reagents were purchased from S.I.A.L. S.r.l. (Rome, Italy).

### 2.10 Cell line authentication and validation

MCF7wt (human female breast adenocarcinoma cells) and U2OS (human female osteosarcoma cells) were obtained from American Type Culture Collection (ATCC, Bethesda, MD) in July 2025. Tumor cells were authenticated by short tandem repeat (STR) profiling using the AmpFℓSTR® NGM Select PCR Amplification Kit followed by capillary electrophoresis analysis. The obtained STR profiles were consistent with the expected MCF7wt and U2OS profiles (≥80%). The cell lines used in this study were constantly monitored and were free from *mycoplasma* contamination during the experiments described in the manuscript.

### 2.11 Cell viability assay

Cell viability was assessed using the MTT assay (3-(4,5-dimethylthiazol-2-yl)-2,5-diphenyltetrazolium bromide; Sigma-Aldrich, Milan, Italy) (Barbanente et al., 2025a). Cells were seeded at a density of 10 × 10^3^ cells per well in 96-well flat-bottom plates, with 50 μL of the test compounds added at final concentrations ranging from 0.1 to 100 μM, bringing the total volume to 100 μL. Untreated cells served as the positive control. After 72 h of incubation at 37 °C in a humidified atmosphere containing 5% CO_2_, MTT was added to each well at a final concentration of 0.5 mg/mL, and cells were incubated for an additional 3–4 h. The culture medium was then removed, and the resulting insoluble formazan crystals were dissolved by adding 100 μL of a 1:1 (v/v) mixture of DMSO and ethanol. Absorbance was measured at 570 nm using a PerkinElmer V3 plate reader. Cell growth inhibition was calculated as the percentage of viable cells relative to the untreated control, and EC_50_ values were determined from dose–response curves using GraphPad Prism version 5.0.

### 2.12 Oxidative stress

Cell viability was assessed using the MTT assay (3-(4,5-dimethylthiazol-2-yl)-2,5-diphenyltetrazolium bromide; Sigma-Aldrich, Milan, Italy) (Barbanente et al., 2025a). Cells were seeded at a density of 10 × 10^3^ cells per well in 96-well flat-bottom plates, with 50 μL of the test compounds added at final concentrations ranging from 0.1 to 100 μM with or without 100 µM α-tocopherol, bringing the total volume to 100 μL. Untreated cells served as the positive control. After 48 h of incubation at 37 °C in a humidified atmosphere containing 5% CO_2_, MTT was added to each well at a final concentration of 0.5 mg/mL, and cells were incubated for an additional 3–4 h. The culture medium was then removed, and the resulting insoluble formazan crystals were dissolved by adding 100 μL of a 1:1 (v/v) mixture of DMSO and ethanol. Absorbance was measured at 570 nm using a PerkinElmer V3 plate reader. Cell growth inhibition was calculated as the percentage of viable cells relative to the untreated control, and EC_50_ values were determined from dose–response curves using GraphPad Prism version 5.0.

### 2.13 *In cellulo* DNA assay

The DNA content of cultured MCF7wt and U2OS was quantified as a measure for cell proliferation using the QuantiFluor™ dsDNA System according to the instructions of the manufacturer. DNA concentrations were normalized against untreated controls. The values are represented as mean standard deviation (n = 3).

### 2.14 *Ex cellulo* DNA assay

The *ex cellulo* DNA interaction with dinuclear platinum complexes was evaluated using the QuantiFluor™ dsDNA System with minor modifications. Different concentrations of dsDNA standard solution (to 0.05 from 5 ng/mL) were incubated with or without 10 µM of dinuclear platinum complexes. After 24, 48, 72 or 168 h of incubation at 37 °C, 10 μL of dsDNA standard solution with or without dinuclear platinum complexes were added to 200 μL of QuantiFluor® dsDNA Dye working solution in 96-well plates black, flat-bottom. The fluorescence (504nmEx/531nmEm) was measured using a plate reader. The values are represented as mean standard deviation (n = 3).

## 3 Results

### 3.1 Synthesis of the dinuclear platinum complexes

The synthesis of the complex [{Pt(1*R*,2*R*-DACH)}_2_(PAM)]NO_3_ (**PtBP3**) was carried out using 2.5 equivalents of [Pt (OSO_3_)(OH_2_)(1*R*,2*R*-DACH)] and 1 mol equivalent of PAM. To study the formation mechanism of **PtBP3**, ^1^H- and ^31^P-NMR spectra were recorded at different time intervals (Figure 2). The ^31^P NMR spectrum, recorded immediately after the addition of the reagents (*ca.* 15 min), shows, in addition to the signal of free PAM (singlet at 17.79 ppm, indicated by a full circle in Figure 2B), a less shielded signal that falls at 27.01 ppm (indicated by a star in Figure 2B). Based on literature data on analogous complexes (Barbanente et al., 2025b), this latter signal can be assigned to the two phosphorus atoms of a mononuclear [Pt (1*R*,2*R*-DACH)(PAM)]^+^ complex (Figure 2A). After 30 min of reaction, two even more deshielded doublets appear, which fall at 39.48 and 38.98 ppm (^2^ *J*_P-P_ = 17.83 Hz), assigned to the two phosphorus atoms of the dinuclear complex (indicated by a triangle in Figure 2B), while the signal of free PAM decreases in intensity. In confirmation of this assignment, the signal associated with the free PAM disappears over time, the signal of the mononuclear complex decreases in intensity, and simultaneously the signal associated with **PtBP3** increases. The coordination of the bisphosphonate to the platinum generally causes the acidification of the reaction solution. Therefore, after 4 h of reaction, the pH was brought to 4 to allow the reaction to reach completion. Indeed, after 18 h of reaction, the only signals in the ^31^P NMR spectrum are those associated with **PtBP3** (Figure 2B), indicating the end of the reaction. To summarize, this study highlighted that the formation of the dinuclear species occurs through the formation of a monomeric Pt-bisphosphonate intermediate complex [Pt(1*R*,2*R*-DACH)(PAM)]^+^ and that an optimal formation of the dinuclear complex is achieved at pH 4, where the reaction reaches completion. Therefore, based on this information, the reaction was carried out on a larger scale in a reaction flask, maintaining the pH at a constant value of 4. To obtain the precipitation of the dinuclear complex in saline form (nitrate as counter-ion), the poor solubility in acetone was exploited by adding an excess of this solvent to the mother liquor after acidification with HNO_3_ to allow protonation of the amino nitrogen of pamidronate.

**FIGURE 2:**
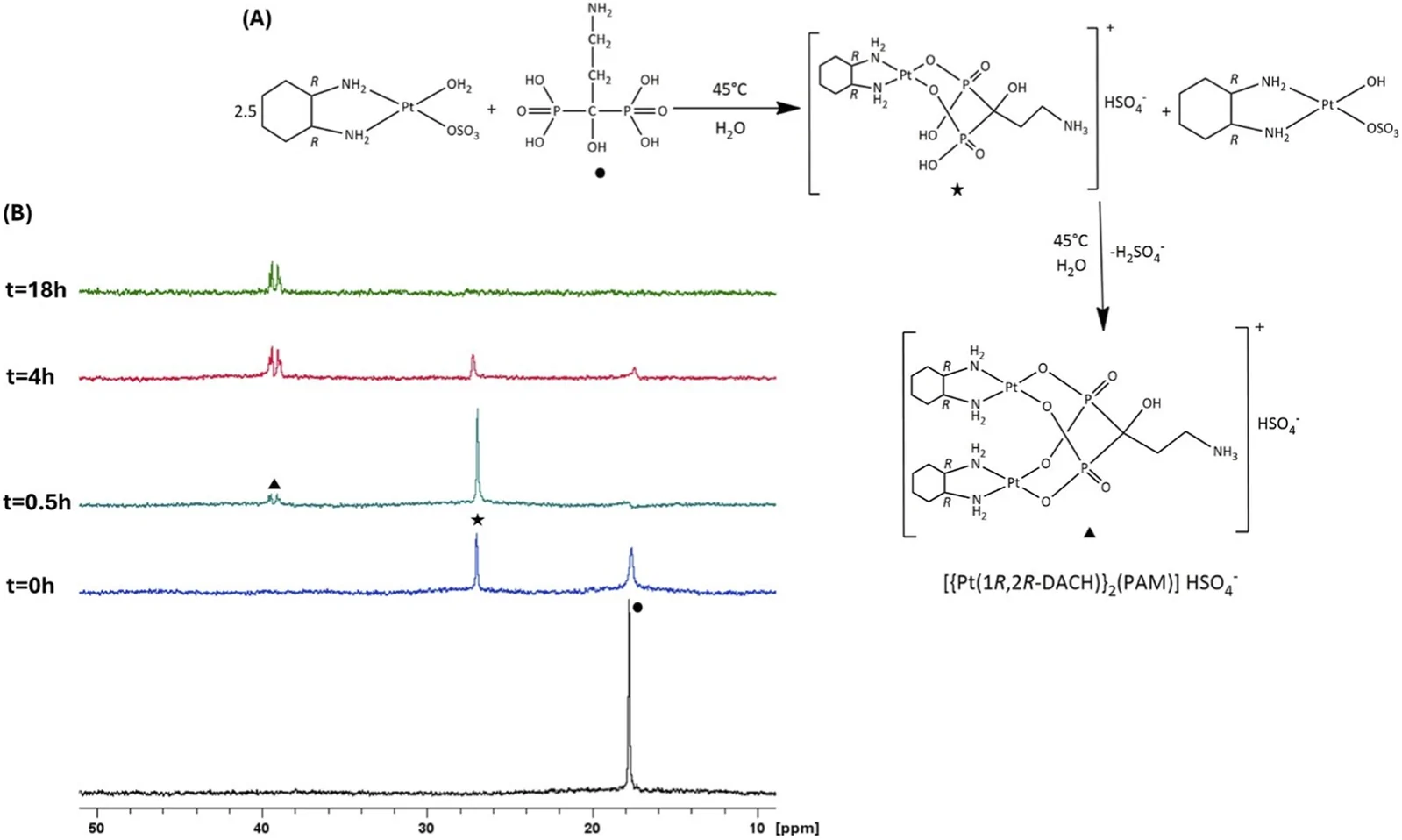
**(A)** Scheme of the mechanism of formation of **PtBP3**. **(B)** ^31^P{^1^H} NMR spectra of the reaction mixture in D_2_O recorded at different time intervals.

Similarly to **PtBP3**, the reaction mechanism of the formation of **PtBP4** was studied in an NMR tube by recording ^31^P NMR spectra at different time intervals (Figure 3). Immediately after the addition of the reagents [Pt(OSO_3_)(OH_2_)(*cis*-1,4-DACH) and PAM, the ^31^P NMR shows both the signal of free BP, which falls at 17.79 ppm (indicated by a circle in Figure 3B), and a deshielded signal at 25.48 ppm (indicated by a star in Figure 3B). The latter signal can be assigned to the two phosphorus atoms of a mononuclear [Pt(*cis*-1,4-DACH)(PAM)]^+^ complex. After *ca.* 30 min of reaction, the appearance of an even more deshielded signal (indicated by a triangle in Figure 3B) is observed and it can be assigned to two phosphorus atoms of a dinuclear complex, while the signal associated with the free PAM disappears. Over time the signal of the mononuclear species decreases in intensity and simultaneously the signal associated with the dinuclear complex increases. After 50 h of reaction, the only signal present in the ^31^P spectrum is that of **PtBP4**, thus indicating the end of the reaction. Also in this case, the formation of the dinuclear complex occurs through the formation of a monomeric Pt-bisphosphonate intermediate complex [Pt (*cis*-1,4-DACH)(PAM)]^+^, which subsequently reacts with a further Pt residue forming the dinuclear complex. Analogously to **PtBP3**, the synthesis of **PtBP4** was carried out on a larger scale in a reaction flask and the dinuclear complex precipitated in saline form by addition of an excess of acetone. Details about NMR characterization of the novel dinuclear Pt-BPs complexes **PtBP3** and **PtBP4**, are shown in Supplementary Figures S1 and S2, respectively.

**FIGURE 3:**
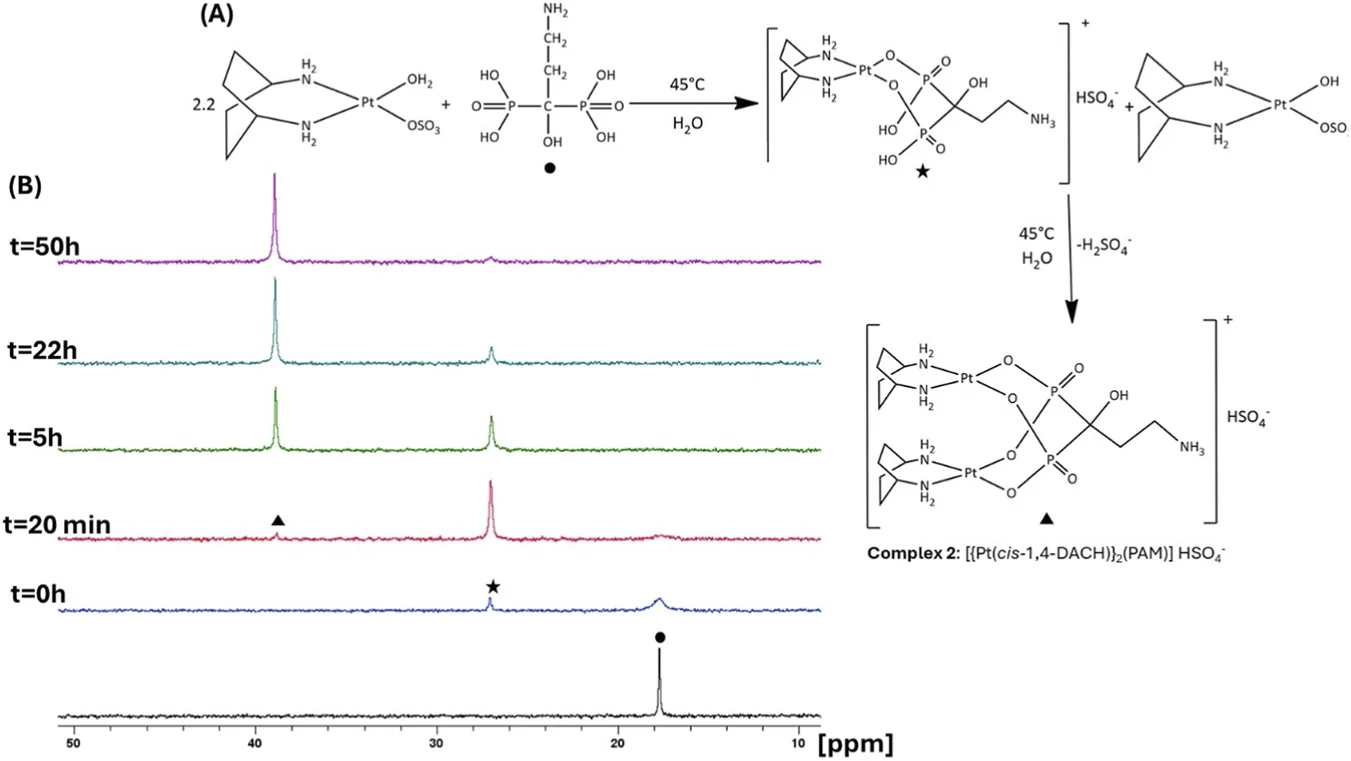
**(A)** Scheme of the mechanism of formation of **PtBP4**. **(B)** ^31^P{^1^H} NMR spectra of the reaction mixture in D_2_O recorded at different time intervals.

### 3.2 Stability in physiological and slightly acidic environment

The stability of **PtBP3** and **PtBP4** under physiological conditions was studied by ^31^P NMR spectroscopy, monitoring the solutions of the two complexes in D_2_O containing HEPES buffer (50 mM, pH = 7.4) and NaCl (120 mM), maintained at 37 °C. The ^31^P NMR spectrum (Figure 4A) of **PtBP3** recorded after 24 h, shows a decrease in the intensity of the signals associated with the initial compound (indicated by a square in Figure 4A; 39.48 and 38.98 ppm) and the simultaneous formation of poorly resolved signals around 27.01 ppm (indicated by a dot in Figure 4A). The latter signal is tentatively attributable to a mononuclear Pt-BP complex [Pt(1*R*,2*R*-DACH)(PAM)]^+^, or to a derivative obtained by the release of a [Pt(1*R*,2*R*-DACH)]^2+^ residue, in agreement with previous ^31^P NMR studies on structurally related Pt–bisphosphonate complexes (Barbanente et al., 2025b).

**FIGURE 4:**
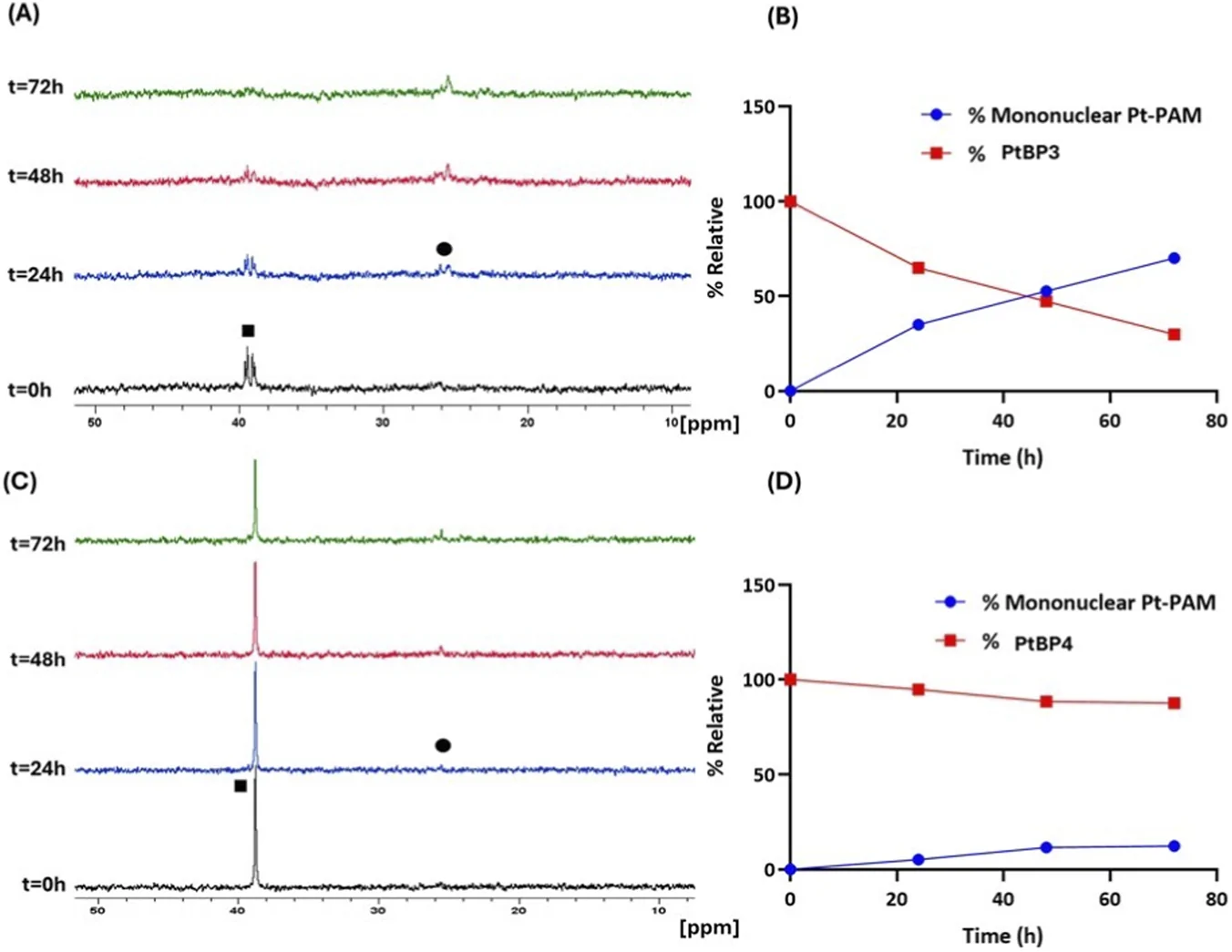
**(A)** ^31^P NMR (121.5 MHz) spectra at different times of **PtBP3** in physiological conditions (D_2_O, HEPES buffer 50 mM, pH* = 7.4, 120 mM NaCl, 37 °C). **(B)** Relative percentages of **PtBP3** and mononuclear Pt-PAM species in physiological conditions, obtained by integration of ^31^P NMR signals, plotted as a function of time. **(C)** ^31^P NMR (121.5 MHz) spectra at different times of **PtBP4** in physiological conditions. **(D)** Relative percentages of **PtBP4** and mononuclear Pt-PAM species in physiological conditions, obtained by integration of ^31^P NMR signals, plotted as a function of time.

After 72 h, the latter signal represents 70.11% of the total phosphorus (Figure 4B). No signal is observed for free PAM (which should be observed, under these experimental conditions, at 17.79 ppm). The stability study in physiologically similar conditions was carried out also for **PtBP4**. We detected that after 72 h (Figure 4C), in addition to the signal attributed to **PtBP4** (indicated with a square; 38.84 ppm), a scarcely visible signal at 25.48 ppm, assigned to the mononuclear species (indicated by a dot) which represents only 12.36% of the total phosphorus (Figure 4D). Also in the case of **PtBP4**, no signal is observed for free PAM (17.79 ppm).

Since it is known that tumor masses are surrounded by a slightly acidic environment (Parks et al., 2013; Webb et al., 2011), the stability of **PtBP3** and **PtBP4** was also studied at 37 °C and pH* = 6.5 by ^31^P NMR spectroscopy, with the aim of verifying whether these complexes would remain stable in these conditions or they could act as prodrugs capable of releasing the cytotoxic Pt portion at the level of the acid tumor mass or bone metastasis. As shown in Figure 5A, the spectrum of **PtBP3** (indicated by a square; 39.48 and 38.98 ppm), recorded after 24 h from dissolution, shows the appearance of a not well-resolved signal falling at 27.01 ppm (indicated by a dot in Figure 5A) assigned to the monomeric Pt-BP complex. This signal, after 72 h represents 48% of the total phosphorus (Figure 5B). Also at acid pH, as already observed at pH* = 7.4, no signal relating to free PAM (17.79 ppm) was detected. Similarly to what observed at pH* = 7.4, **PtBP4** (Figure 5C; indicated by a square; 38.84 ppm) is found to be more stable; indeed, at pH* = 6.5, after 72 h from dissolution, the mononuclear species (indicated by a dot; 25.48 ppm), represents only 13.4% of the total phosphorus (Figure 5D). Therefore, even under weak acidic conditions, **PtBP4** turns out to be more stable than **PtBP3**.

**FIGURE 5:**
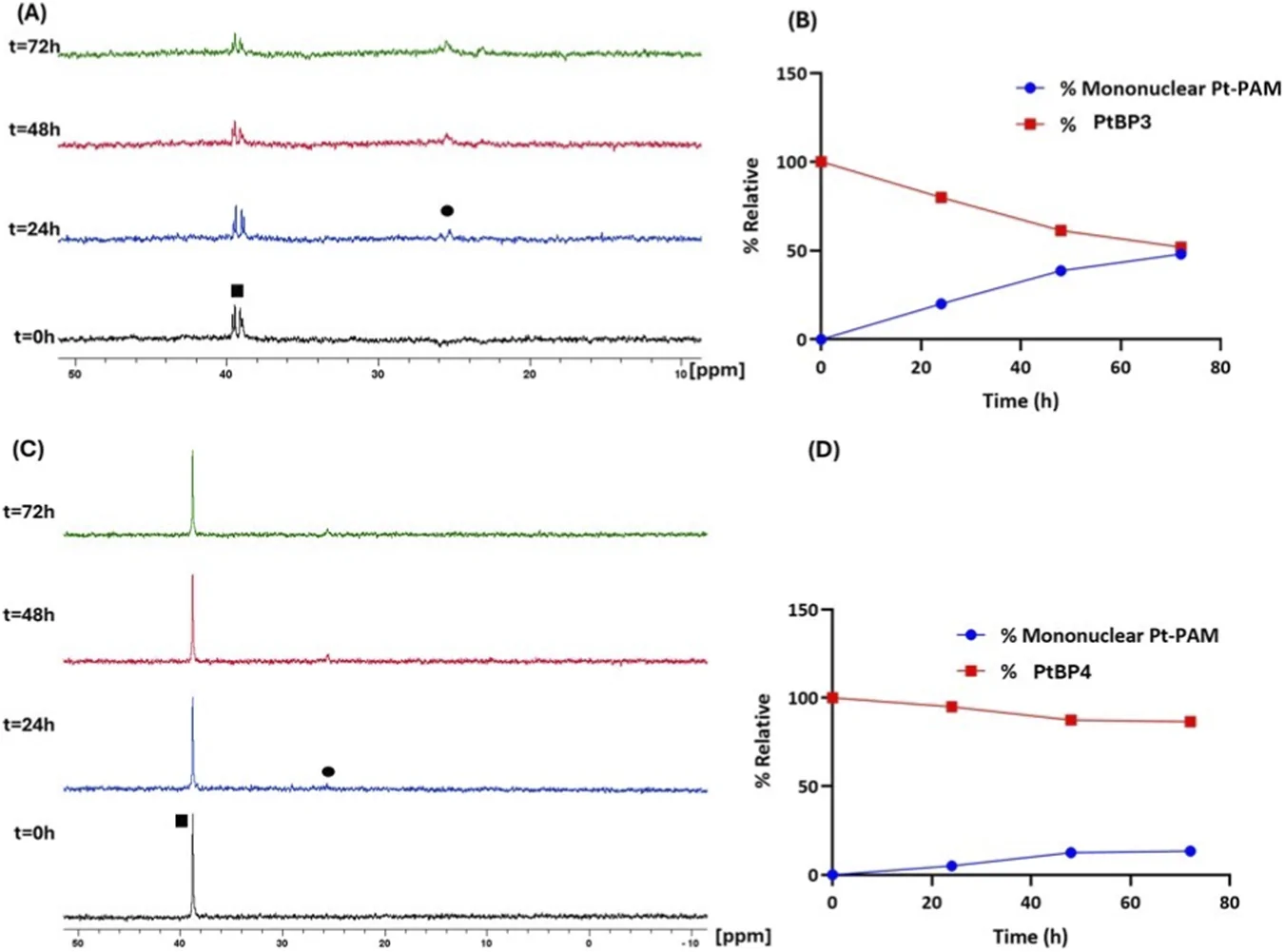
**(A)** ^31^P NMR (121.5 MHz) spectra at different times of **PtBP3** in acidic conditions (D_2_O, HEPES buffer 50 mM, pH* = 6.5, 120 mM NaCl, 37 °C). **(B)** Relative percentages of **PtBP3** and mononuclear [Pt(1*R*,2*R*-DACH)(PAM)]^+^ in acidic conditions, obtained by integration of ^31^P NMR signals, plotted as a function of time. **(C)** ^31^P NMR (121.5 MHz) spectra at different times of **PtBP4** in acidic conditions. **(D)** Relative percentages of **PtBP4** and mononuclear [Pt(*cis*-1,4-DACH)(PAM)]^+^ species in acidic conditions, obtained by integration of ^31^P NMR signals, plotted as a function of time.

### 3.3 Reactivity with 5’-GMP investigated by multinuclear NMR

An important aspect of Pt-based drugs is their ability to interact with DNA by forming coordination bonds with the N7 of guanine, thus leading to the formation of Pt-DNA adducts. Therefore, 5’-GMP (5’-guanosine monophosphate) is often used as the simplest model of DNA to preliminarily study the mechanism of interaction between platinum complexes and DNA. The reaction of complexes **PtBP3** and **PtBP4** with 5’-GMP (4.5 equivalents, 0.011 mmol) was therefore studied in an NMR tube in 50 mM HEPES buffer (pH* = 7.4) and 120 mM NaCl at 37 °C by ^31^P and ^1^H NMR spectroscopy. In the reaction of complex **PtBP3**, the ^31^P NMR spectrum recorded immediately after the addition of the reagents, shows the signal of free 5’-GMP (4.09 ppm; indicated by a dot in Figure 6B and the signals assigned to the dinuclear complex (39.48 and 38.98 ppm; indicated with a triangle). The ^31^P NMR recorded after 24 h of reaction shows a new signal at 4.21 ppm, which can be reasonably assigned (Barbanente et al., 2025b) to the 5’-GMP phosphorous of the bis-adduct [Pt(5’-GMP)_2_(1*R*,2*R*-DACH)] (indicated by an hexagon in Figures 6A,C). Furthermore, the spectrum shows the appearance of two new signals that fall, respectively, at 25.95 and 25.31 ppm which can be assigned to the monomeric Pt-BP complex observed in the stability study performed at pH 7.4 (see Figure 6; indicated with a star in Figure 6B). The two signals at 25.95 and 25.31 ppm were assigned to the two magnetically nonequivalent phosphorus nuclei of the mononuclear Pt–BP species, resulting from the absence of a symmetry plane passing through the BP geminal carbon atom, the Pt center, and the DACH carrier ligand. A^31^P spectrum recorded after 96 h of reaction at a higher field (500 MHz, ^1^H) (Figure 6E), allowed the detection of a further signal, resonating at 3.55 ppm, attributable to the phosphorus atoms of 5’-GMP of the mono-adduct [Pt(5’-GMP)X(1*R*,2*R* -DACH) (X = Cl or H_2_O) indicated with a diamond in Figure 6E.

**FIGURE 6:**
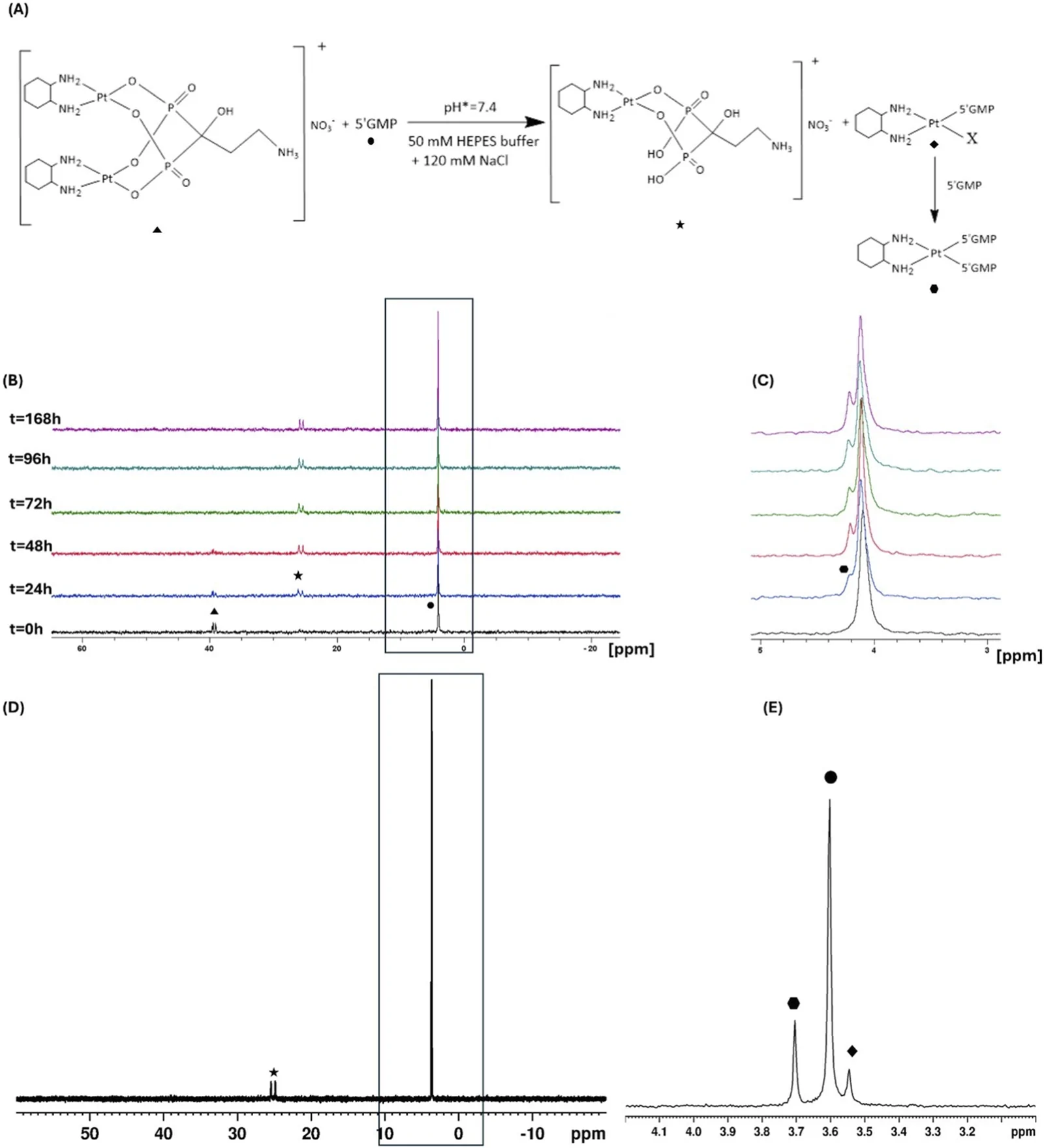
**(A)** Reaction scheme of complex **PtBP3** with 5’-GMP. **(B)** ^31^P NMR spectra of **PtBP3** with 5’-GMP at different time intervals (300 MHz,^1^H). **(C)** Magnification of the box indicated in **(B)**. **(D)** ^31^P NMR spectra after 96 h of reaction (500 MHz, 1H). **(E)** Enlargement of the box indicated in **(D)**.

The reactivity of **PtBP3** with 5’-GMP was also monitored by recording ^1^H NMR spectra (Figure 7A). The spectrum recorded at t = 0 h shows only the signals belonging to free 5’-GMP: a singlet at 8.20 ppm and a doublet at 5.92 ppm (indicated by a dot in Figure 7) belonging, respectively, to the protons H_8_ and H_1’_ of guanine. After 24 h, the spectrum shows two new sets of signals falling at 8.83 and 5.98 (indicated with a diamond in Figure 7A) and 8.59 and 5.87 ppm (indicated by a hexagon in Figure 7A), respectively. In particular, the set of signals resonating at 8.83 and 5.98 ppm can be confidentially assigned to the protons H_8_ and H_1’_ of the coordinated 5’-GMP in the mono-adduct [Pt(5’-GMP)X (1*R*-2*R*-DACH)] (X = Cl or H_2_O) (Ranaldo et al., 2008), while the signals at 8.59 and 5.87 ppm are assigned, respectively, to the protons H_8_ and H_1’_ of the two coordinated 5’-GMP residues in the bis-adduct [Pt(5’-GMP)_2_(1*R*,2*R*-DACH)]. The intensity of the two new sets of signals increases progressively over time, while at the same time the intensity of the signals associated with free 5’-GMP decreases. By recording the ^1^H NMR spectrum at higher field (Figure 7B), the signals appear more clearly visible and resolved. Data obtained by ^1^H NMR spectroscopy are fully consistent with those obtained by ^31^P NMR spectroscopy.

**FIGURE 7:**
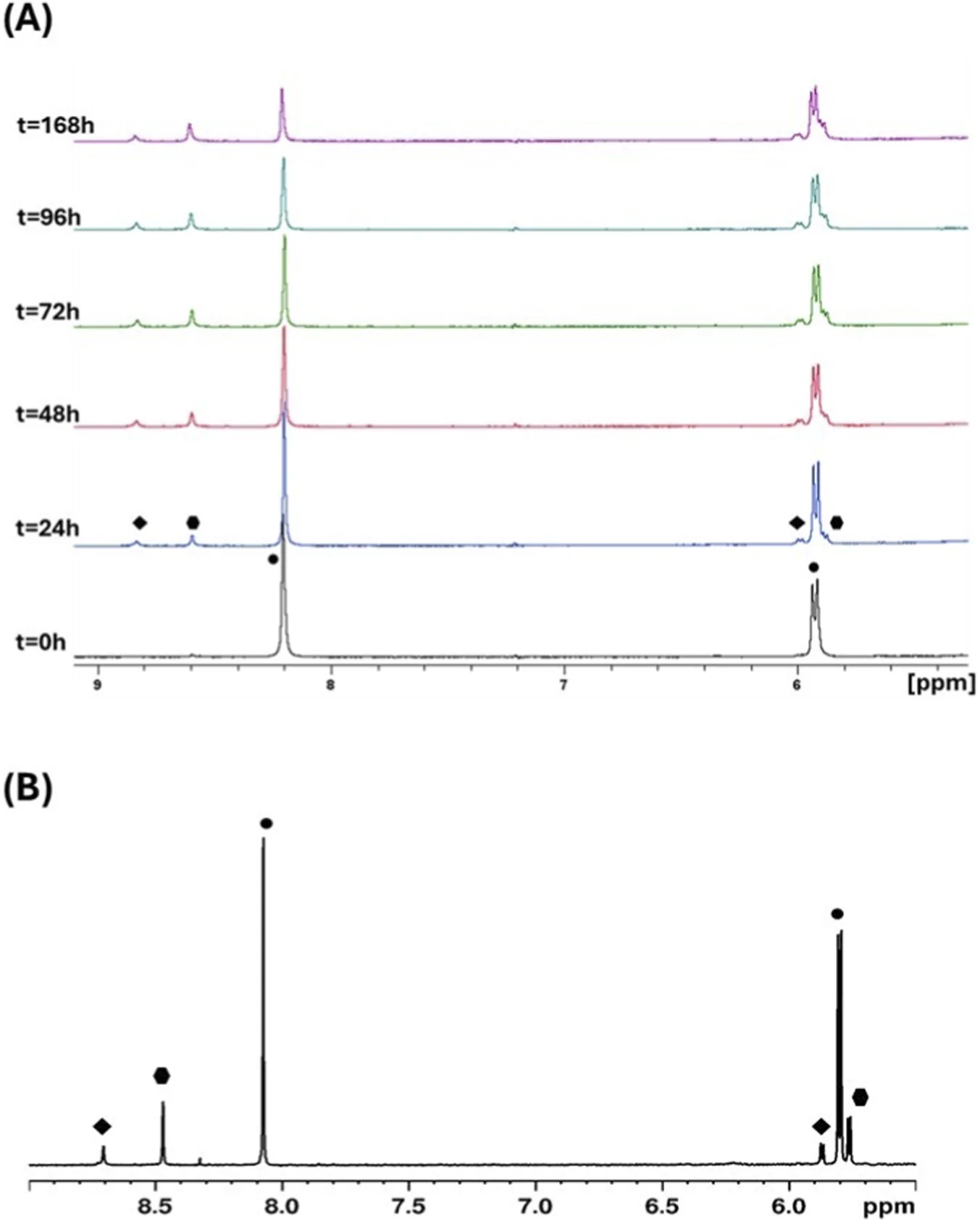
**(A)** ^1^H NMR spectra of the reaction between **PtBP3** and 5’-GMP recorded at different time intervals (300 MHz, ^1^H). **(B)** ^1^H NMR spectrum recorded after 96 h of reaction at higher field (500 MHz, ^1^H).

Also in the case of **PtBP4**, the reactivity with 5’-GMP was monitored by recording ^31^P (Supplementary Figure S3) and ^1^H (Supplementary Figure S4) NMR spectra.

### 3.4 Reactivity with other DNA models investigated by HRMS

The interaction of **PtBP3** and **PtBP4** with two distinct DNA models was further investigated using high-resolution mass spectrometry (HRMS). Our goal was to directly demonstrate the formation of platinum-DNA adducts, a key mechanism underlying their biological activity. First, we employed 9-Ethylguanine (9-EtG) as a simplified DNA base model to monitor adduct formation (at N7 of guanine). This approach parallels the previous NMR studies done with 5’-GMP and is supported by our initial findings (Barbanente et al., 2025b; Getreuer et al., 2024). The compounds were incubated with 9-EtG at 37 °C for time intervals of 24 h and 7 days, followed by infusion into the mass spectrometer for analysis. Remarkably, after just 24 h, both compounds **PtBP3** and **PtBP4** formed clear adducts with 9-EtG (Figure 8A; Supplementary Figure S5, respectively), thereby corroborating prior NMR results. Notably, the observed adducts involved coordination with two 9-EtG molecules, suggesting that upon ligand release, these platinum complexes can potentially cross-link DNA strands either inter- or intra-molecularly. Next, we examined interactions with a biologically relevant oligonucleotide derived from the promoter region of the oncogene c-MYC, a sequence capable of folding into a G-quadruplex structure. G-quadruplexes are secondary DNA structures increasingly recognized for their role in cancer biology and gene regulation. This oligonucleotide contains nucleobases accessible for platinum(II) coordination. Incubations with both compounds were carried out under identical conditions (37 °C, 24 h and 7 days). High-resolution mass spectra revealed distinct peaks corresponding to the five-charge state of unmodified c-Myc with two NH_4_^+^ ions (observed *m/z* 1,404.0439; calculated *m/z* 1,404.0424, Supplementary Figure S6), as well as peaks representing the **PtBP3**-c-Myc adduct (observed *m/z* 1,458.6479; calculated *m/z* 1,458.6446, Figure 8B) after 24 h, confirming formation of a stable adduct after the release of the two NH_4_^+^ ions. These data strongly indicate a robust interaction between **PtBP3** and the G-quadruplex DNA. In both 9-EtG and c-Myc cases, adduct formation is consistent with coordination of the mononuclear platinum species [Pt(1*R*,2*R*-DACH)]^2+^ following ligand release from the original complexes. In contrast, no corresponding adduct peaks were detected for the compound **PtBP4**-c-Myc incubation at either 24 h or 7 days, underscoring the lower reactivity of **PtBP4** toward this DNA structure under the tested conditions. These findings collectively demonstrate the capability of these platinum-based compounds, particularly compound **PtBP3**, to form covalent adducts with DNA models ranging from simple bases to biologically significant G-quadruplex structures, providing insights into their modes of anticancer action.

**FIGURE 8:**
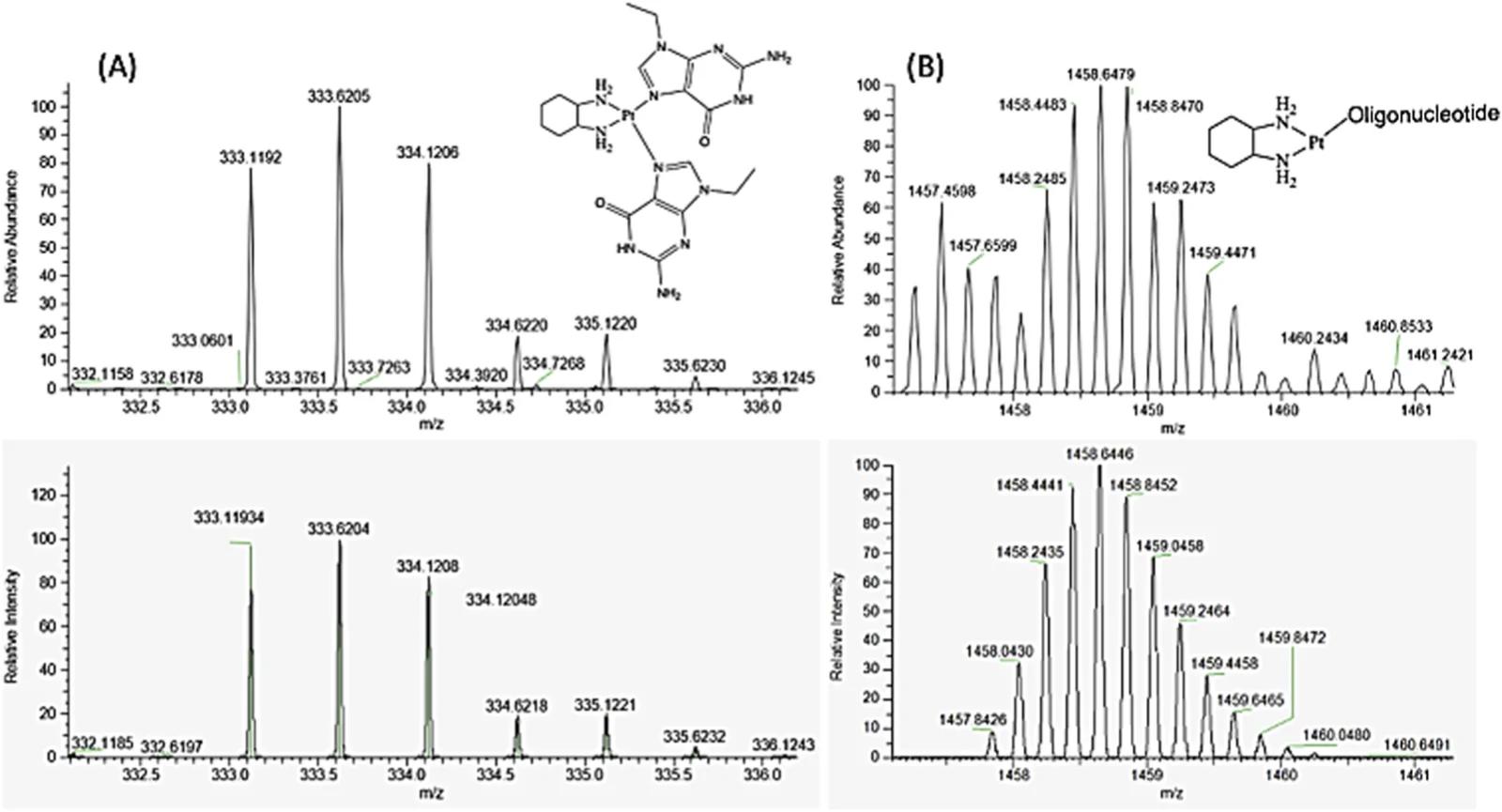
HRMS of compound **PtBP3** incubated with **(A)** 9-EtG and **(B)** c-Myc for 24 h at 37 °C. Adduct of **PtBP3** with two 9-EtG: found: 333.6205, calculated: 333.6205 (M for C_20_H_32_N_12_O_2_Pt, charge 2+). Adduct of **PtBP3** with c-Myc oligonucleotide: found: 1458.6479, calculated: 1458.6446 (M−H for C_226_H_282_N_97_O_131_P_21_Pt, charge 5-).

### 3.5 Direct *ex-cellulo* interaction with dsDNA investigated by fluorescence assays

To extend our analysis beyond mononucleotides and oligonucleotide models, we investigated the direct interaction of the PAM-bridged dinuclear platinum complexes with free double-stranded DNA using an *ex-cellulo* QuantiFluor™ dsDNA fluorescence assay. In parallel, the analogous zoledronate-bridged complexes were included in this study (**PtBP1** and **PtBP2**) as their reactivity toward DNA models has been previously characterized and shown (Barbanente et al., 2025b). In this experiment, a fixed concentration of each complex (10 μM) was incubated with increasing concentrations of free dsDNA, 0–2000 ng/mL, corresponding to the range used to generate the standard calibration curve. Fluorescence emission of the DNA–dye adduct was monitored over an extended time course of up to 7 days, matching the timescale employed for the 5′-GMP reactivity experiments performed in NMR tubes. This parallel timing allows direct comparison between the kinetics of DNA model adduct formation in the NMR tube and the evolution of fluorescence signals in the *ex-cellulo* assay. A time-dependent decrease in dsDNA fluorescence was observed for all four complexes see (Figure 9). After 24 h of incubation, all complexes produced a detectable reduction in dsDNA-associated fluorescence relative to the DNA control. At this point, the decrease was already more pronounced for the 1*R*,2*R*-DACH-containing complexes **PtBP1** and **PtBP3**, whereas the *cis*-1,4-DACH analogues **PtBP2** and **PtBP4** showed a more modest effect. After 72 h, the time-dependent trend was fully established for all four compounds, with **PtBP1** and **PtBP3** consistently producing the strongest fluorescence quenching across the entire dsDNA concentration range, and **PtBP2** and **PtBP4** displaying intermediate behaviour. This pattern was preserved after 7 days of incubation, with no inversion or saturation effects observed, indicating a sustained interaction between the complexes and dsDNA over the monitored period. Overall, the fluorescence profiles reveal clear and persistent carrier-dependent behavior, in line with the relative reactivity previously observed for these complexes toward DNA model systems (5′-GMP, 9-ethylguanine, and c-Myc G-quadruplex).

**FIGURE 9:**
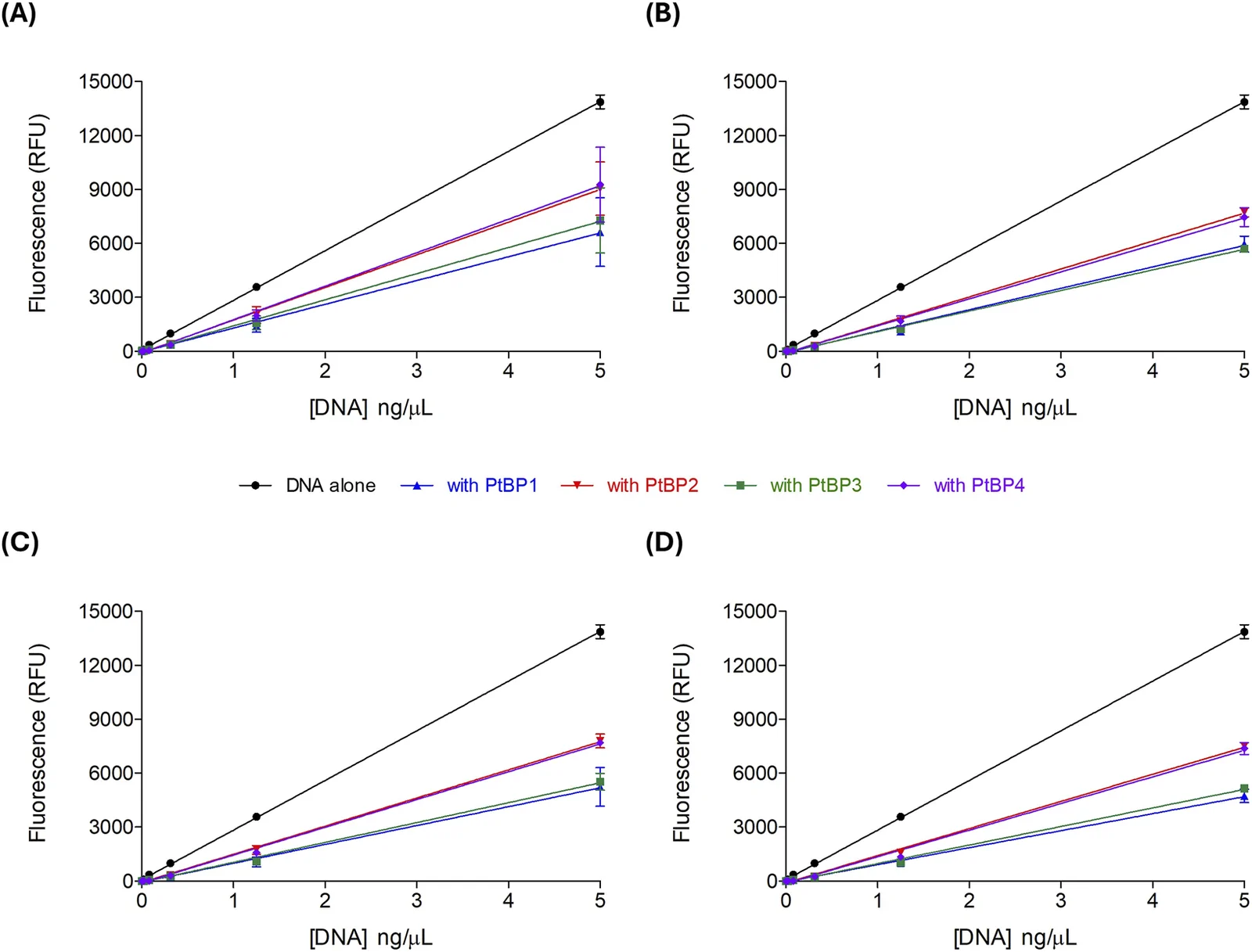
*Ex-cellulo* interaction of **PtBP1-4** with free double-stranded DNA evaluated using the QuantiFluor™ dsDNA fluorescence assay. Fluorescence was measured after **(A)** 24 h, **(B)** 48 h, **(C)** 72 h, and **(D)** 168 h. All curves are compared with that of untreated DNA. Data represent mean values from three independent experiments.

### 3.6 Biological evaluation

#### 3.6.1 *In vitro* cytotoxicity

The cytotoxic activity of **PtBP3** and **PtBP4** was assessed in MCF7wt and U2OS cells after 72 h of incubation using the MTT assay, with different concentrations of the tested compounds. Alongside U2OS osteosarcoma cells, MCF7wt breast cancer cells were included in the study since breast carcinoma is among the malignancies that most frequently metastasize to bone, making both models relevant for evaluating the potential activity of bisphosphonate-containing platinum complexes. The corresponding EC_50_ values, calculated from dose-survival curves, are reported in Table 1. The values for **PtBP1**, **PtBP2**, oxaliplatin and zoledronic acid in MCF7wt cells were previously published by some of us in an earlier study (Barbanente et al., 2025b) and are included here for comparative purposes. All EC_50_ values reported for U2OS, as well as those for the PAM derivatives **PtBP3** and **PtBP4** in both cell lines, were determined in the present work for the first time. The antiproliferative activity of all complexes was compared with the corresponding platinum drugs and the free bisphosphonates, evaluated under the same experimental conditions after 72 h of incubation. In MCF7wt cells, the previously published data showed that the 1*R*,2*R*-DACH complex **PtBP1** possessed a lower EC_50_ value than its *cis*-1,4-DACH analogue **PtBP2**. A comparable trend was observed for the PAM derivatives, with **PtBP3** exhibiting stronger cytotoxicity than **PtBP4** (12.5 and 32.5 μM, respectively). In U2OS cells, a similar carrier-dependent pattern was obtained: **PtBP1** and **PtBP3** produced the lowest EC_50_ values within their respective bisphosphonate families (7.1 and 10.3 μM, respectively), whereas **PtBP2** and **PtBP4** showed higher values under the same conditions (30.7 and 56.4 μM, respectively). Furthermore, the ZL-containing complexes (**PtBP1** and **PtBP2**) displayed lower EC_50_ values than the corresponding PAM derivatives, indicating higher overall cytotoxic activity under the tested conditions. In general, across both cell lines, the relative ranking of cytotoxicity remains consistent, with the 1*R*,2*R*-DACH derivatives (**PtBP1** and **PtBP3**) systematically more active than their *cis*-1,4-DACH analogues (**PtBP2** and **PtBP4**). Oxaliplatin and kiteplatin were included as reference compounds for the 1*R*,2*R*-DACH and *cis*-1,4-DACH carrier ligands, respectively, while zoledronic acid and pamidronate were tested as free bisphosphonates. In both MCF7wt and U2OS tumor cells, kiteplatin showed higher cytotoxic activity than oxaliplatin. The free bisphosphonates zoledronic acid and pamidronate did not show an antiproliferative activity in MCF7wt cells under the same experimental conditions, while a conjugation of the bisphosphonate to platinum fragments resulted in a substantial enhancement of cytotoxicity compared to the free bisphosphonates alone, indicating that the observed antiproliferative effects are primarily driven by the platinum moieties. In U2OS cells, both oxaliplatin and kiteplatin retained measurable cytotoxic activity, and a noticeable antiproliferative effect was also observed for pamidronate and zoledronic acid. In this context, it is worth noting that zoledronic acid has been reported to exert direct antiproliferative and pro-apoptotic effects in human osteosarcoma cell lines *in vitro*, suggesting that nitrogen-containing bisphosphonates may display intrinsic cytotoxic activity beyond their bone-resorption inhibition properties (Evdokiou et al., 2003). Nevertheless, all PtBP complexes exhibited higher EC_50_ values in U2OS than in MCF7wt cells, indicating an overall lower sensitivity of the osteosarcoma model to these treatments, while preserving the same carrier-dependent trend observed in breast cancer cells.

**TABLE 1:** Antiproliferative activity (EC_50_, μM) obtained by the MTT test after 72 h of incubation with different concentrations of the tested compounds. Data are expressed as mean ± standard error of the mean (SEM) of three independent experiments each performed in triplicate.

Antiproliferative effect (EC_50_ μM ± SEM)		
Compounds	MCF7wt	U2OS
PtBP1	9.12*	7.1 ± 0.5
PtBP2	27.2*	30.7 ± 5.0
PtBP3	12.5 ± 2.1	10.3 ± 0.9
PtBP4	32.5 ± 2.7	56.4 ± 4.3
Oxaliplatin	30.6*	8.34 ± 2.2
Kiteplatin	5.4 ± 0.8	2.36 ± 0.3
Zoledronic acid	n.a*	<1
Pamidronic acid	n.a	3.12 ± 0.5

* From reference (Barbanente et al., 2025b); n.a. = not active.

#### 3.6.2 *In-cellulo* interaction with dsDNA

The effect of the PAM-containing complexes **PtBP3** and **PtBP4** on the cellular DNA content was evaluated in MCF7wt and U2OS cells using the QuantiFluor™ dsDNA System after 48 h of incubation (Figure 10). This assay provides a quantitative measure of the amount of double-stranded DNA remaining in the cell population following treatment across the 1.56–100 μM concentration range. In both cell lines, exposure to increasing concentrations of the two complexes resulted in a progressive decrease in the dsDNA signal. **PtBP3** induced a stronger reduction in DNA content compared to **PtBP4** across the tested concentration range, in line with the carrier-dependent differences observed in the MTT assay. In MCF7wt cells, the decrease in dsDNA fluorescence became evident already at intermediate concentrations, where cell viability dropped below 40% (Figure 10C), whereas in U2OS cells the reduction appeared more gradual, although still dose-dependent for both compounds (Figure 10D). For comparison, the corresponding zoledronate-containing complexes **PtBP1** and **PtBP2** were evaluated under the same conditions (Figures 10A,B). Both complexes reduced dsDNA fluorescence in a concentration-dependent manner in MCF7wt and U2OS cells. As observed for **PtBP3** and **PtBP4**, the oxaliplatin derivative **PtBP1** produced a greater decrease in DNA content than its kiteplatin analogue **PtBP2** in both cell lines. In MCF7wt, **PtBP1** induced a marked reduction of the dsDNA signal already at the lower concentrations tested (Figure 10A), where cell viability decreased to approximately 50%, while **PtBP2** showed a more moderate decrease. In U2OS, both complexes produced a dose-dependent reduction, although the decrease in dsDNA fluorescence remained less pronounced than in MCF7wt (Figure 10B).

**FIGURE 10:**
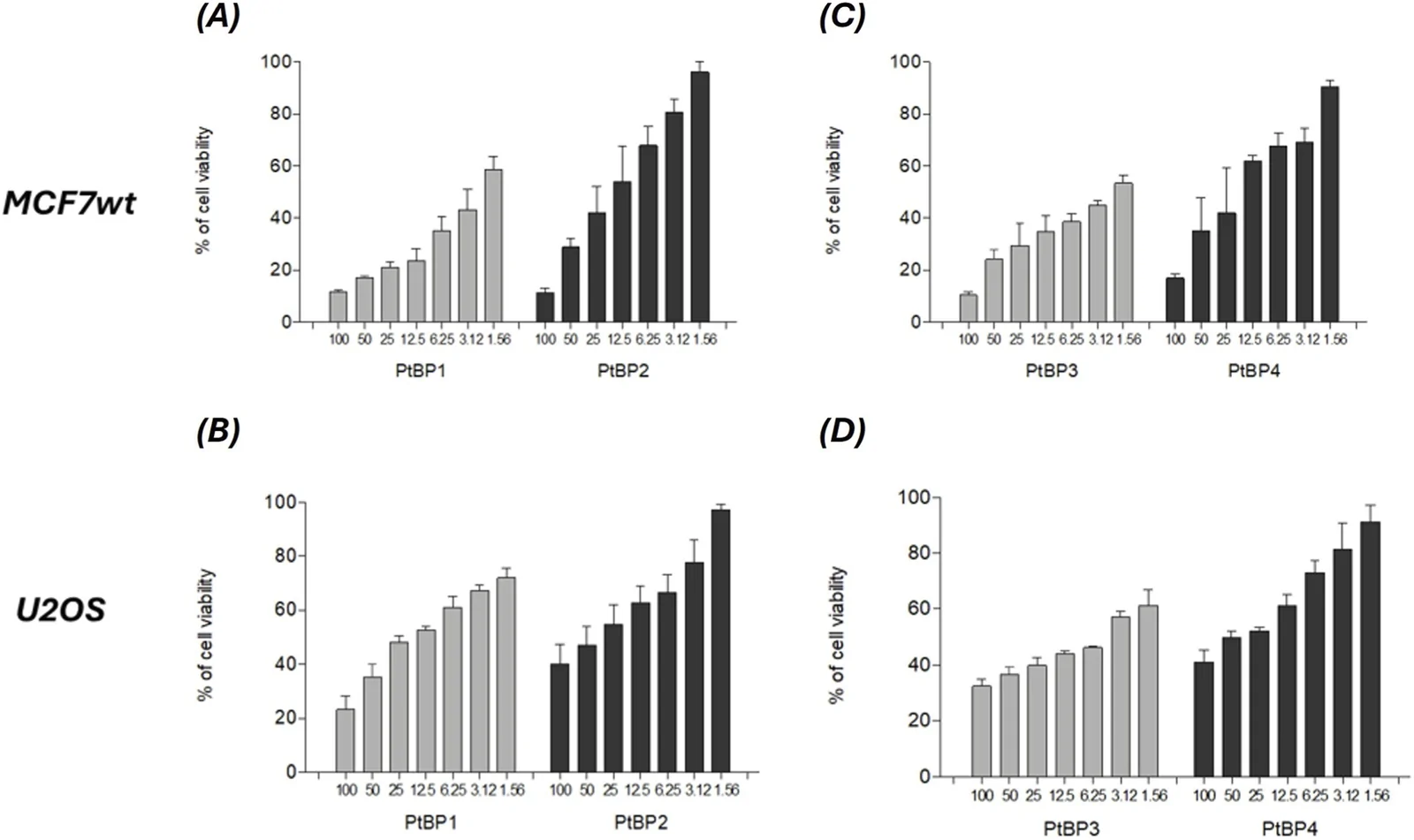
Effect of PtBP complexes on cellular DNA content in MCF7wt and U2OS cells after 48 h of treatment, quantified using the QuantiFluor™ dsDNA System. **(A,B)** Zoledronate-bridged complexes. **(C,D)** Pamidronate-bridged complexes. The dsDNA fluorescence signal, expressed as percentage relative to untreated controls, decreases progressively with increasing concentrations of the complexes (1.56–100 μM), reflecting reduced intracellular double-stranded DNA content. Data represent mean ± SD of three independent experiments.

#### 3.6.3 Oxidative stress

To explore the possibility of a secondary mechanism contributing to the cytotoxicity of these novel dinuclear Pt-BPs complexes, beyond the canonical DNA-targeting pathway typical of platinum(II) drugs, the MTT assay was also performed in the presence of the antioxidant α-tocopherol (100 μM) after 48 h of incubation in both MCF7wt and U2OS cells, in the 1.56–100 μM concentration range (Figure 11). Co-treatment with α-tocopherol was used to determine whether partial rescue of cell viability could be observed under antioxidant conditions, since oxidative stress is a well-known contributor to cancer cell death, particularly through ROS-induced damage to cellular structures such as lipids, proteins, and DNA (Zhao et al., 2023). In MCF7wt (Figure 11), co-treatment with α-tocopherol (100 μM) produced a clear dose-dependent rescue of cell viability for both **PtBP3** and **PtBP4**. In particular, for **PtBP3**, at the highest concentrations (50–100 μM), cell viability increased from 55% to 85% at 50 μM and from 35%–40% to 65%–70% at 100 μM, indicating a substantial partial reversal of cytotoxicity. A similar, although slightly attenuated, trend was observed for **PtBP4**. In U2OS cells, the overall cytotoxic effect of both complexes was less pronounced, and the impact of α-tocopherol, as a consequence, was reduced.

**FIGURE 11:**
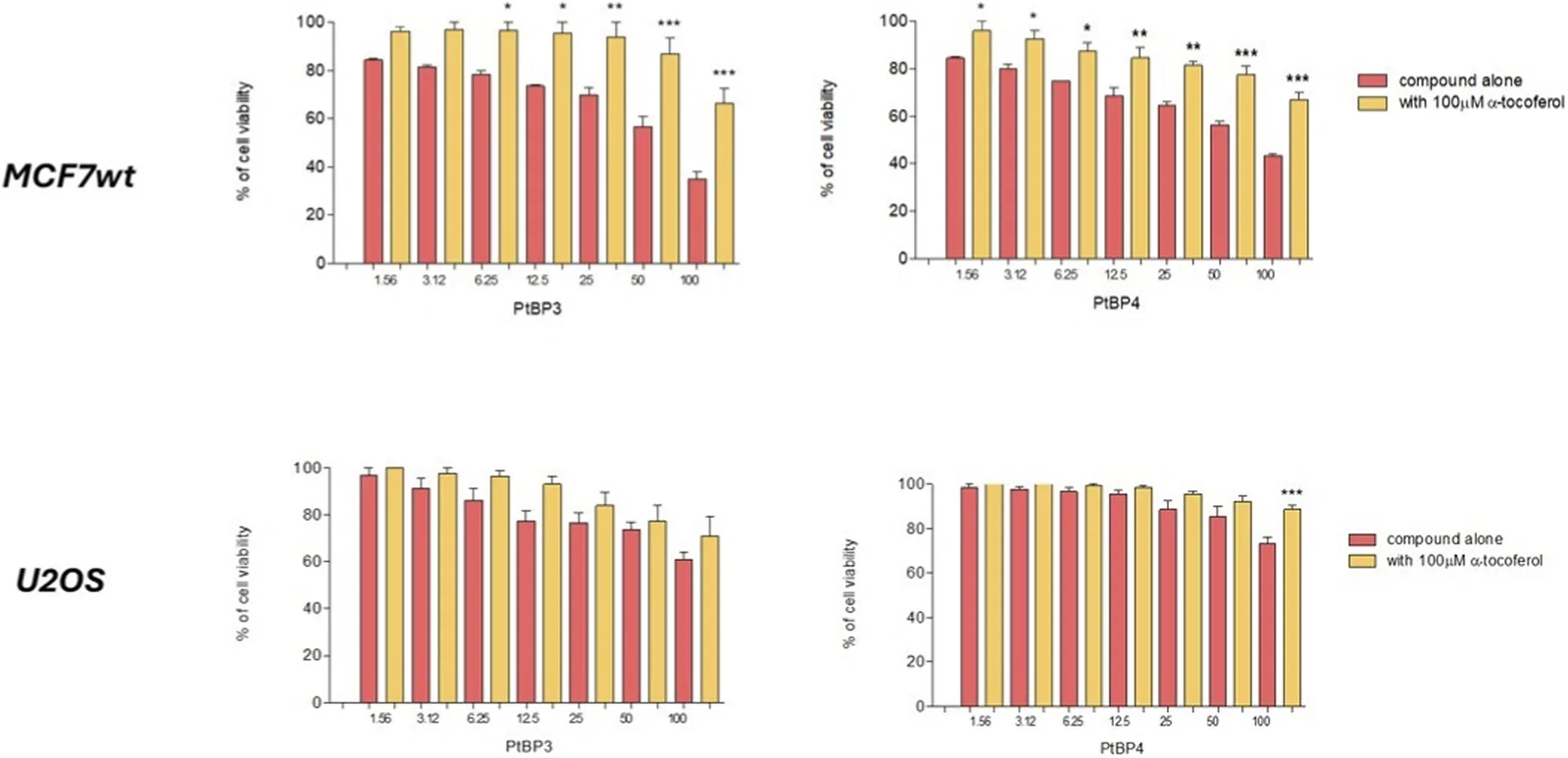
Effect of α-tocopherol on the cytotoxicity of PAM-containing dinuclear platinum complexes in MCF7wt and U2OS cells after 48 h of incubation. Cell viability was assessed by the MTT assay over the 1.56–100 µM concentration range. Data are expressed as mean ± SD of three independent experiments. Statistical significance was calculated relative to treatment with the compound alone (*p < 0.05, **p < 0.01, **p < 0.001).

The overall activity pattern mirrors what was recently reported by some of us for the corresponding zoledronate-containing complexes, where the involvement of ROS in MCF7wt cytotoxicity was demonstrated (Barbanente et al., 2026). In the present work, this assessment is also extended to U2OS cells (Figure 12). Also in this case, the oxaliplatin derivative **PtBP1** was the most cytotoxic, while the rescue induced by α-tocopherol appeared more pronounced in MCF7wt than in U2OS. In order to assess whether prolonged exposure could enhance the cytotoxic effect and the rescue of cell viability, particularly in U2OS cells, the results of MTT with and without α-tocopherol obtained after 72 h of incubation are reported in the SI (Supplementary Figure S7). Under these longer exposure conditions, the same activity trend was maintained: the 1*R*,2*R*-DACH complexes displayed a stronger cytotoxic effect than the corresponding *cis*-1,4-DACH analogues in both cell lines, and the rescue induced by α-tocopherol remained more pronounced in MCF7wt than in U2OS. These data suggest that, in addition to the primary cytotoxic pathway, the antiproliferative activity of the investigated complexes may involve an oxidative component that can be modulated by antioxidant exposure.

**FIGURE 12:**
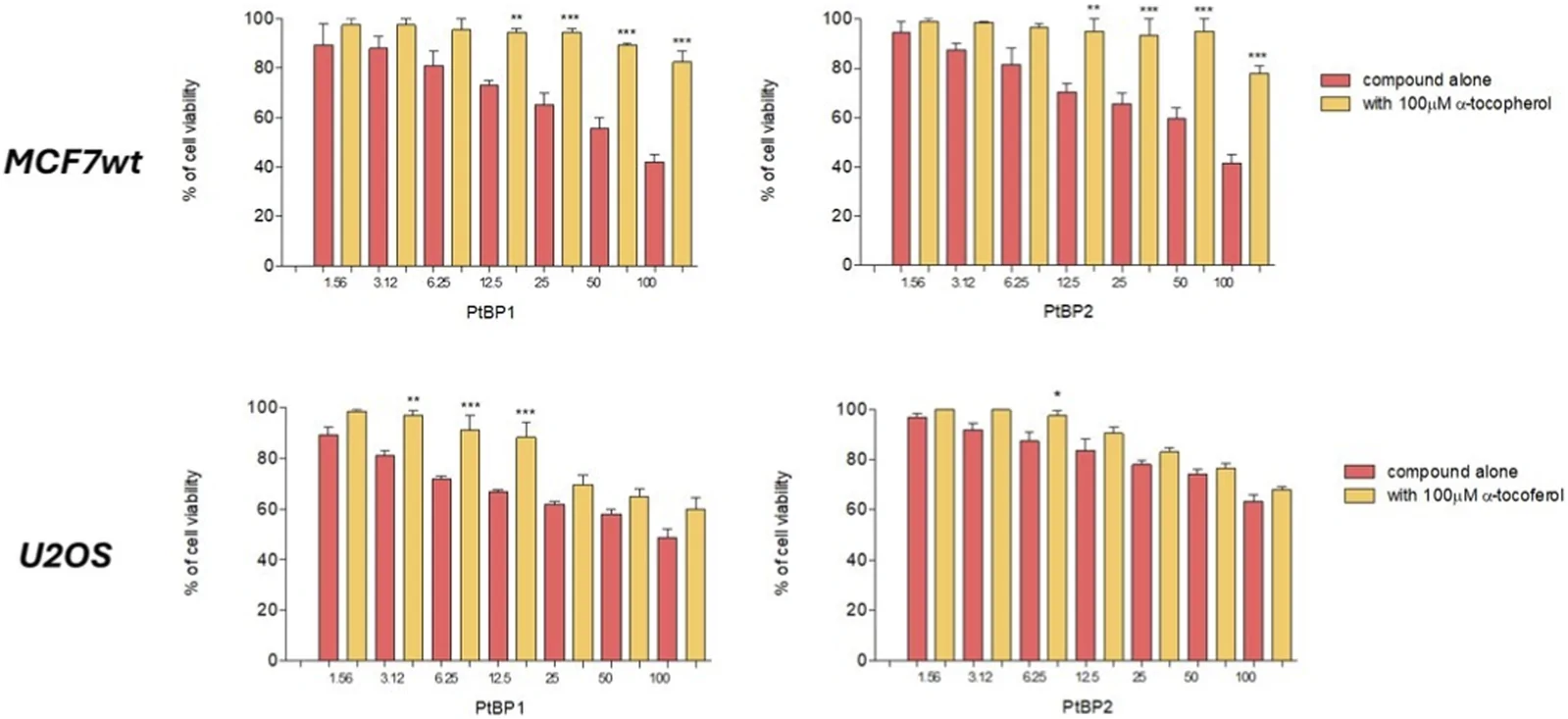
Effect of α-tocopherol on the cytotoxicity of the zoledronate-bridged dinuclear platinum complexes **PtBP1** and **PtBP2** in MCF7wt and U2OS cells after 48 h of incubation. The MCF7wt data for **PtBP1** and **PtBP2** were previously reported (Barbanente et al., 2026) and are shown here for comparison, while the U2OS data were obtained in the present study. Statistical significance was calculated relative to treatment with the compound alone (*p < 0.05, **p < 0.01, **p < 0.001).

## 4 Discussion

### 4.1 Cell-type and bisphosphonates dependence of cytotoxic responses

The EC_50_ values derived from the MTT assays provide a quantitative measure for comparing the antiproliferative profiles of the investigated complexes across the two cellular models. All dinuclear PtBP complexes exhibited EC_50_ values in the micromolar range, with slightly lower values observed in MCF7wt cells than in U2OS cells, indicating a higher sensitivity of the breast cancer model. Within both cell lines, the 1*R*,2*R*-DACH (oxaliplatin) derivatives systematically displayed lower EC_50_ values than their *cis*-1,4-DACH (kiteplatin) analogues, confirming the dominant role of the carrier ligand in governing cytotoxic potency. In contrast, the free bisphosphonates did not show an antiproliferative activity in MCF7wt cells, whereas a measurable cytotoxic effect was observed in U2OS cells, consistent with literature reports indicating that nitrogen-containing bisphosphonates may exert intrinsic antiproliferative activity beyond their antiresorptive function (Evdokiou et al., 2003). Comparison between the two cellular models further highlights a clear cell-type dependence, with U2OS cells displaying higher EC_50_ values, reduced DNA perturbation and a more limited antioxidant-mediated rescue, suggesting a lower overall susceptibility to these treatments. Finally, comparison between PAM and ZL containing complexes indicates that the nature of the bisphosphonate modulates the overall cytotoxicity without altering the carrier-dependent structure–activity relationship. ZL derivatives (**PtBP1** and **PtBP2**) exhibited lower EC_50_ values than their PAM analogues in both cell lines, in agreement with their higher reactivity reported previously (Barbanente et al., 2025b). The lower cytotoxic potency of the PAM-containing complexes reflects the role of several factors rather than differences in chemical stability alone. Within the PAM series, the higher activity of **PtBP3** compared to **PtBP4** parallels its greater lability and higher reactivity toward DNA models, suggesting that a more rapid generation of reactive Pt species may contribute to its enhanced cytotoxicity. However, this relationship does not fully account for the differences between the PAM- and ZL-containing derivatives, as **PtBP3** remains less active than its ZL analogue **PtBP1**. The nature of the bisphosphonate scaffold may therefore influence additional parameters governing cellular activity, including complex speciation, DNA reactivity, cellular uptake, and intracellular platinum accumulation. Since cellular uptake and intracellular Pt levels were not directly investigated in the present study, their contribution cannot be established and warrants further investigation. Nevertheless, PAM-containing complexes retained the same qualitative behavior, including enhanced activity of the 1*R*,2*R*-DACH derivatives, significant effects on cellular DNA, and partial rescue by α-tocopherol. These findings indicate that replacing the bisphosphonate does not alter the fundamental biological behavior of this class of complexes, while allowing the design of species with potential bone-targeting activity. These structure–activity relationships could also provide a basis for future computationally assisted optimization of Pt–BP complexes, as artificial intelligence, machine-learning, molecular modeling, and virtual screening approaches are increasingly used in medicinal chemistry to predict biological activity and drug–target interactions, explore chemical space, and guide the design and prioritization of new anticancer candidates with improved physicochemical and biological properties (Mullowney et al., 2023; Xi et al., 2021; Zhavoronkov et al., 2026). Overall, both PAM and ZL containing complexes show strong and consistent activity, with the carrier ligand being the main factor influencing their reactivity and cytotoxic effects.

### 4.2 Influence of the carrier ligand on chemical stability

In this work we examined the stability of the complexes under both physiological and mildly acidic conditions in order to better understand their solution behavior and the factors that may influence their activity in biological environments. The stability of **PtBP3** and **PtBP4** under physiological conditions was studied by ^31^P NMR spectroscopy, monitoring the solutions of the two complexes in buffered D_2_O. The stability differences observed between **PtBP3** and **PtBP4** under both physiological and slightly acidic conditions can be rationalized by considering the distinct geometries adopted by their diamine carrier ligands. Under physiological conditions (pH 7.4), **PtBP4** exhibited markedly higher stability than **PtBP3**, with only a small fraction of mononuclear complex detected after 72 h. In contrast, **PtBP3**, after 72 h, shows a signal attributable to the mononuclear species that represents the majority of the total phosphorus. Interestingly, both complexes showed increased stability at mildly acidic pH (pH 6.5), although the overall stability ranking was maintained, with **PtBP4** remaining significantly more stable than **PtBP3**. The enhanced stability of **PtBP3** under acidic conditions may be relevant in the context of tumor microenvironments or bone metastases, where slightly acidic conditions are typically present. Interestingly, the greater stability of **PtBP4** compared with **PtBP3** may also contribute to its lower cytotoxic activity. A slower formation of reactive platinum species from **PtBP4** could limit or delay their interaction with intracellular DNA, whereas the greater lability of **PtBP3** may facilitate the generation of DNA-reactive species, consistently with its higher reactivity toward DNA models and greater cytotoxicity. Based on previous data (Barbanente et al., 2025b), we hypothesize that the stability of these complexes under the two investigated conditions (physiological and acidic) depends on the different geometry of the DACH ring. In brief, in **PtBP3**, the diamine ligand is nearly co-planar to the coordination plane of the metal (Johnstone, 2014) while in **PtBP4**, *cis*-1,4-DACH adopts a boat conformation, that is perpendicular to the coordination plane of the metal (Ranaldo et al., 2008). This geometry introduces a greater steric hindrance along the *z*-axis, where ligand substitution would typically occur. However, further studies are needed to confirm this hypothesis.

### 4.3 Influence of the carrier ligand on chemical behavior and DNA reactivity

Given that the anticancer efficacy of platinum-based agents is largely attributed to their ability to form coordination adducts with DNA, particularly through binding to the N7 position of guanine, we investigated the reactivity of the complexes toward a series of DNA models of increasing structural complexity. As a first approach, 5′-GMP was employed as the simplest mononucleotide model to monitor the formation of Pt–guanine adducts by ^31^P and ^1^H NMR spectroscopy under physiological conditions. To further assess their interaction with nucleic acid targets, the complexes were examined by high-resolution mass spectrometry using additional DNA models, including an oligonucleotide and the c-Myc G-quadruplex–forming sequence. Finally, to extend our analysis to free double-stranded DNA and complement the data obtained from model systems, we evaluated the direct interaction of the complexes with purified dsDNA using an ex-cellulo QuantiFluor™ fluorescence assay. The results obtained for the two novel Pt-BPs complexes (**PtBP3** and **PtBP4**) clearly highlighted the key role played by the carrier ligand in governing both the chemical reactivity and the biological profile of the dinuclear platinum complexes. The 1*R*,2*R*-DACH derivative **PtBP3** consistently displayed higher reactivity than its *cis*-1,4-DACH analogue **PtBP4** in all chemical assays performed, including the formation of adducts with 5′-GMP, 9-ethylguanine, and the c-Myc G-quadruplex sequence. Nevertheless, it is important to note that, unlike the analogous dinuclear complex [{Pt(1*R*,2*R*-DACH)}_2_(ZL)]NO_3_ (ZL = zoledronate) investigated in the previous study (Barbanente et al., 2025b), [{Pt(1*R*,2*R*-DACH)}_2_(PAM)]NO_3_ or **PtBP3**, despite sharing the same carrier ligand, exhibits a slightly different behavior. In the compound containing zoledronate, free zoledronate is detected after 168 h of reaction, indicating a complete hydrolysis of the initial dinuclear Pt-BP complex. The overall results of the study of the reactivity towards the simplest model of DNA 5’-GMP showed that both platinum(II) dinuclear complexes (**PtBP3** and **PtBP4**) react with 5'-GMP forming the adduct [Pt(5'-GMP)_2_(A_2_)]^+^ (A_2_ = 1*R*,2*R*-DACH or *cis*-1,4-DACH), through the reaction of [Pt(A)_2_]^2+^ (released by hydrolysis of the complexes) with 5'-GMP. In particular, in the case of **PtBP4**, it was possible to observe the formation of the monoadduct only after 168 h of reaction. In conclusion, **PtBP3** was found to be slightly more reactive than complex **PtBP4** towards the DNA model 5'-GMP, likely due to the influence of the carrier ligand. This finding is indeed in line with our previous results, where the reactivity towards 5'-GMP was assessed in analogous complexes containing zoledronate as a bisphosphonate (Barbanente et al., 2025b). The greater reactivity of the 1*R*,2*R*-DACH complexes is further supported by the *ex-cellulo* fluorescence assay using free double-stranded DNA, where **PtBP3** produced a stronger and more rapid decrease in fluorescence than **PtBP4**. Taken together, these observations indicate that the coordination environment imposed by the 1*R*,2*R*-DACH ligand favors interaction with DNA, whereas the *cis*-1,4-DACH analogue results in slower adduct formation. This marked dependence on the carrier ligand also provides a rationale for further structural optimization of the DACH scaffold. Structure–activity relationship studies represent a valuable approach for guiding the structural optimization of anticancer candidates and identifying modifications associated with improved biological properties (Qin et al., 2024; Qiu et al., 2021). Previous structure–activity relationship studies on oxaliplatin analogues have shown that alkyl substitution of the DACH ring, including the introduction of a methyl group at the 4-position, can significantly modulate anticancer activity, with the stereochemistry of the substituent influencing the biological response (Jungwirth et al., 2012). Accordingly, selected modifications of the DACH ligand could be explored in future PtBP derivatives to further modulate their chemical reactivity and biological activity. More broadly, modification of the bisphosphonate scaffold and the development of Pt(IV) derivatives may provide additional strategies to optimize the physicochemical and pharmacological properties of these complexes.

### 4.4 Contribution of DNA damage to cytotoxic activity

To better understand the contribution of DNA damage to the cytotoxic effects of the complexes, we assessed how **PtBP3** and **PtBP4** alter the cellular DNA content using the QuantiFluor™ dsDNA assay, and compared these findings with those obtained for the ZL containing PtBP complexes whose synthesis and characterization were recently reported by some of us (Barbanente et al., 2025b). This approach provides a quantitative measure of cellular dsDNA content in treated MCF7wt and U2OS cells. In addition to U2OS osteosarcoma cells, MCF7wt breast cancer cells were included due to the high incidence of bone metastases associated with breast carcinoma, to provide two biologically relevant models for studying bisphosphonate-containing platinum complexes. The results obtained from the *in-cellulo* DNA content assay support the relevance of DNA as a biological target for the novel PtBPs complexes. All complexes induced a dose-dependent decrease in dsDNA fluorescence in both MCF7wt and U2OS cells, consistent with the formation of DNA adducts that lead to the apoptosis of the treated cells. In agreement with the chemical reactivity studies, the 1*R*,2*R*-DACH complexes **PtBP1** and **PtBP3** produced a more substantial reduction in DNA content than the *cis*-1,4-DACH analogues. The fact that this trend persists in both PAM and ZL containing complexes suggests that the carrier ligand, rather than the bisphosphonate moiety, is the main determinant of the extent of DNA perturbation. Although both cell lines showed a reduction in dsDNA content, the decrease was generally more pronounced in MCF7wt cells. U2OS cells, while responsive to the treatments, displayed a more gradual reduction in dsDNA fluorescence. The lower cellular response observed in U2OS cells, despite the pronounced interaction of **PtBP1** and **PtBP3** with isolated dsDNA, indicates that intrinsic DNA reactivity alone cannot fully account for the cell-dependent activity of these complexes. Indeed, while *ex-cellulo* experiments directly probe the interaction of the complexes with accessible DNA, cellular activity is influenced by additional processes, including cellular uptake and efflux, intracellular platinum accumulation and distribution, nuclear accessibility, and DNA damage processing and repair (Liang et al., 2025; Slika et al., 2024). Notably, nucleotide excision repair has been shown to modulate the sensitivity of U2OS cells to platinum drugs, including cisplatin and oxaliplatin (Graf et al., 2011). Since these parameters were not directly investigated in MCF7wt and U2OS cells in the present study, their contribution to the different responses of the 2 cell lines remains to be established. Comparative assessment of cellular platinum uptake and intracellular accumulation, together with DNA damage processing and repair, will therefore represent important aspects of future investigations.

### 4.5 Contribution of oxidative stress as a secondary mechanism

To assess whether mechanisms other than direct formation of DNA adducts contribute to the cytotoxicity of these novel dinuclear PtBP complexes, we examined how their antiproliferative effects were modulated by co-treatment with the antioxidant α-tocopherol. This approach allows evaluation of the potential involvement of oxidative stress, which may act in parallel with the canonical DNA-targeting mechanism of platinum(II) agents. All complexes exhibited at least partial rescue of metabolic activity in the presence of the antioxidant, indicating that oxidative stress contributes to their antiproliferative effects. The importance of the rescue was consistently greater for the 1*R*,2*R*-DACH derivatives **PtBP1** and **PtBP3**, which also displayed the highest cytotoxicity and DNA-reactivity profiles, suggesting that oxidative stress may act in coordination with the DNA targeting mechanism. The strength of rescue effect differed between the 2 cell lines. In MCF7wt cells, co-treatment with α-tocopherol produced a marked shift in the dose–response curves, whereas in U2OS cells the rescue effect was more limited. These observations suggest that MCF7wt and U2OS cells may differ in their susceptibility to oxidative stress-mediated damage and in the relative contribution of oxidative stress to their overall cytotoxic response. Differences in cellular redox homeostasis and antioxidant defense mechanisms are known to influence the response of cancer cells to oxidative stress and platinum-based treatments (Gorrini et al., 2013). In particular, the Nrf2–GCLC/GSH pathway has been shown to modulate antioxidant capacity and cisplatin sensitivity in MCF7 cells (Syu et al., 2016). Accordingly, the different extent of α-tocopherol-mediated rescue observed in MCF7wt and U2OS cells may reflect differences in their redox balance or a different contribution of oxidative stress relative to other cytotoxic mechanisms. However, since baseline ROS levels, intracellular glutathione content, and antioxidant pathway activity were not directly assessed in the present study, the molecular basis of this differential response cannot be established. Overall, the partial rescue of cell viability by α-tocopherol supports the hypothesis that oxidative stress may represent an additional mechanism alongside the primary DNA-directed activity of these complexes.

## 5 Conclusion

In this study, two new dinuclear platinum(II) complexes having pamidronate as a bridging ligand (**PtBP3** and **PtBP4**) were synthesized, characterized, and compared with their previously reported zoledronate analogues. NMR investigation of their formation mechanism suggested a stepwise assembly process involving mononuclear Pt–BP intermediates before the generation of the final dinuclear complexes. Chemical reactivity studies showed that the 1*R*,2*R*-DACH derivatives consistently interact faster with DNA models than the *cis*-1,4-DACH complexes, while stability assays highlighted a higher stability for **PtBP4** under both physiological and mildly acidic conditions. Biological evaluation in MCF7wt and U2OS cells confirmed a clear carrier-dependent trend, with the 1*R*,2*R*-DACH complexes (oxaliplatin derivatives) displaying greater cytotoxicity and a stronger impact on cellular DNA content. Co-treatment with α-tocopherol partially rescued cell viability, supporting the hypothesis that oxidative stress may contribute as a secondary mechanism alongside DNA damage. Overall, replacing zoledronate with pamidronate preserves the biological profile of these dinuclear platinum complexes while providing an alternative bisphosphonate for potential bone-targeted applications. These findings highlight the central role of the carrier ligand in shaping both chemical behaviour and cytotoxic activity. Further studies should directly assess the hydroxyapatite-binding properties of the PAM-containing complexes **PtBP3** and **PtBP4**, to complement the hydroxyapatite-binding data previously obtained for the ZL-containing analogues **PtBP1** and **PtBP2** and to better define their bone-targeting potential. Comparative assessment of cellular platinum uptake and intracellular accumulation will also be required to clarify the cell-dependent activity of these complexes. Key translational aspects, including solubility and formulation, pharmacokinetic properties, biodistribution, potential toxicity, and *in vivo* antitumor efficacy, will also need to be investigated. In parallel, further structural optimization of the Pt–BP scaffold may provide opportunities to improve its physicochemical and pharmacological properties. In this context, we are currently investigating the oxidation of these Pt(II) complexes to Pt(IV), with the aim of obtaining more stable prodrug derivatives, improving their physicochemical properties, including solubility, and exploring their potential for oral administration.

## Data Availability

The original contributions presented in the study are included in the article/Supplementary Material, further inquiries can be directed to the corresponding authors.
