# Supplementary material for: Active targeting towards bone cancer and metastases by pamidronate-bridged dinuclear platinum(II) complexes
Source: Front Chem. 2026 Sep 22;14:1945537. doi: 10.3389/fchem.2026.1945537 (PMC13638515; doi:10.3389/fchem.2026.1945537)
Supplement: Supplementary file 1 [file DataSheet1.pdf]

Electronic Supplementary Information

Active targeting towards bone cancer and metastases by Pamidronate-bridged dinuclear platinum(II) complexes

Anna Maria Di Cosola[1], Alessandra Barbanente[1], Luisa D'Anna[2], Simona Rubino[2], Alessio Terenzi[2], Mauro Niso[3*], Nicola Margiotta[1*].

[1]Dipartimento di Chimica, Università degli Studi di Bari Aldo Moro, Via E. Orabona 4, 70125 Bari, Italy.

[2]Dipartimento di Scienze e Tecnologie Biologiche Chimiche e Farmaceutiche, Università degli Studi di Palermo, Viale delle Scienze, Ed. 17, 90128 Palermo, Italy.

[3]Dipartimento di Farmacia-Scienze del Farmaco, Università degli Studi di Bari Aldo Moro, Via E. Orabona 4, 70125 Bari, Italy.

Characterization of [{Pt(1R,2R-DACH)}$_2$(PAM)]NO_3 (PtBP3)

PtBP3 was characterized by elemental analysis, ESI-MS, and ^{1}H,^{31} P,^{195}Pt, [^{1}H,^{1}H]-COSY, [^{1}H,^{13}C]-HSQC and [^{1}H,^{13}C]-HMBC NMR spectroscopy. The ESI-MS(+) spectrum showed the presence of a peak at *m/z* = 850.0885, corresponding to [**PtBP3**+H]$^+$, with an isotopic pattern in excellent agreement with the theoretical one (data not shown). The elemental analysis is also in agreement with the presence in the complex of pamidronate and [Pt(1*R*,2*R*-DACH)]$^{2+}$ residues in a 1:2 ratio. The [^{1}H-^{1}H]-COSY spectrum of **PtBP3** in D_2O (Figure S1A) shows a cross-peak at 3.29/2.21 ppm (A in Figure S1A), assigned to the protons H(3')/H(2'). The cross-peak observed at 2.31/2.53 ppm (B) is instead assigned to the protons H(1)/H(2). Furthermore, the cross-peak falling at 2.31/1.34 ppm (C) is assigned to the protons H(1)/H(3/6ax). The cross-peaks observed at 1.34/2.09 and 1.17/1.62 ppm (D and E, respectively) are assigned to the protons H(3/6ax)/H(3/6eq) and H(4/5ax)/H(4/5eq). Finally, the amino protons of 1*R*,2*R*-DACH are observed in the range 6.5-5.0 ppm. The data obtained did not allow a complete assignment of the amino protons. The ^{31}P NMR spectrum of **PtBP3** in D_2O (Figure S1E) shows two doublets ($^2J_{P\text{-}P}$ = 17.83 Hz) which fall at 39.48 and 38.98 ppm, respectively, assigned to the two phosphorus atoms of the bisphosphonate. The two P atoms are not magnetically equivalent due to the presence of the chiral carbon atoms in the carrier ligand 1*R*,2*R*-DACH and resonate at lower field ($\Delta\delta$ = 21.49 ppm) with respect to those of the free ligand (pamidronate). The ^{195}Pt NMR spectrum of complex **PtBP3** (Figure S1D) shows a broad signal falling at -1668.71 ppm. The structure in which a bisphosphonate unit bridges two platinum atoms in a defined W-shaped conformation, would suggest the presence of two signals in the ^{195}Pt spectrum. The absence of a symmetry plane passing through the PCP atoms (due to the presence of two different substituents on the central carbon atom, the OH and the aminoethyl group) makes the two Pt atoms non-equivalent. However, under the experimental conditions used in our experiment, the two platinum atoms have practically coincident chemical shifts giving rise to a single broad signal. The NMR characterization of **PtBP3** was completed by recording a [^{1}H,^{13}C]-HSQC spectrum in order to assign the chemical shift of the carbon atoms (Figure S1B). The cross-peaks falling at 68/2.31 (A) and 65.61/2.53 (B) (^{13}C/^{1}H) ppm are

respectively assigned to C(1)H(1) and C(2)H(2). The cross-peak at 38.78/3.29 ($^{13}C/^{1}H$) ppm (C) is assigned to C(3')/H(3'). The cross-peaks falling at 34.68/2.09 and 34.68/1.34 ($^{13}C/^{1}H$) ppm (D and E in Fig. 2B) are respectively assigned to C(3/6)(3/6eq) and C(3/6)/H(3/6ax). The cross-peak at 33.89/2.21 ($^{13}C/^{1}H$) ppm (F) is assigned to C(2')/H(2'). Finally, the cross-peaks at 26.93/1.62 (G) and 26.93/1.17 (H) ($^{13}C/^{1}H$) ppm were respectively assigned to C(4/5)/H(4/5eq) and C(4/5)/H(4/5ax). In order to assign the quaternary C(1') ^{13}C chemical shift, an [^{1}H,^{13}C]-HMBC spectrum was recorded (Figure S1C), which presents two cross-peaks falling at 79.82/3.29 (a) and 79.82/2.21 (b) ($^{13}C/^{1}H$) ppm, respectively assigned to C(1')/H(3') and C(1')/H(2').

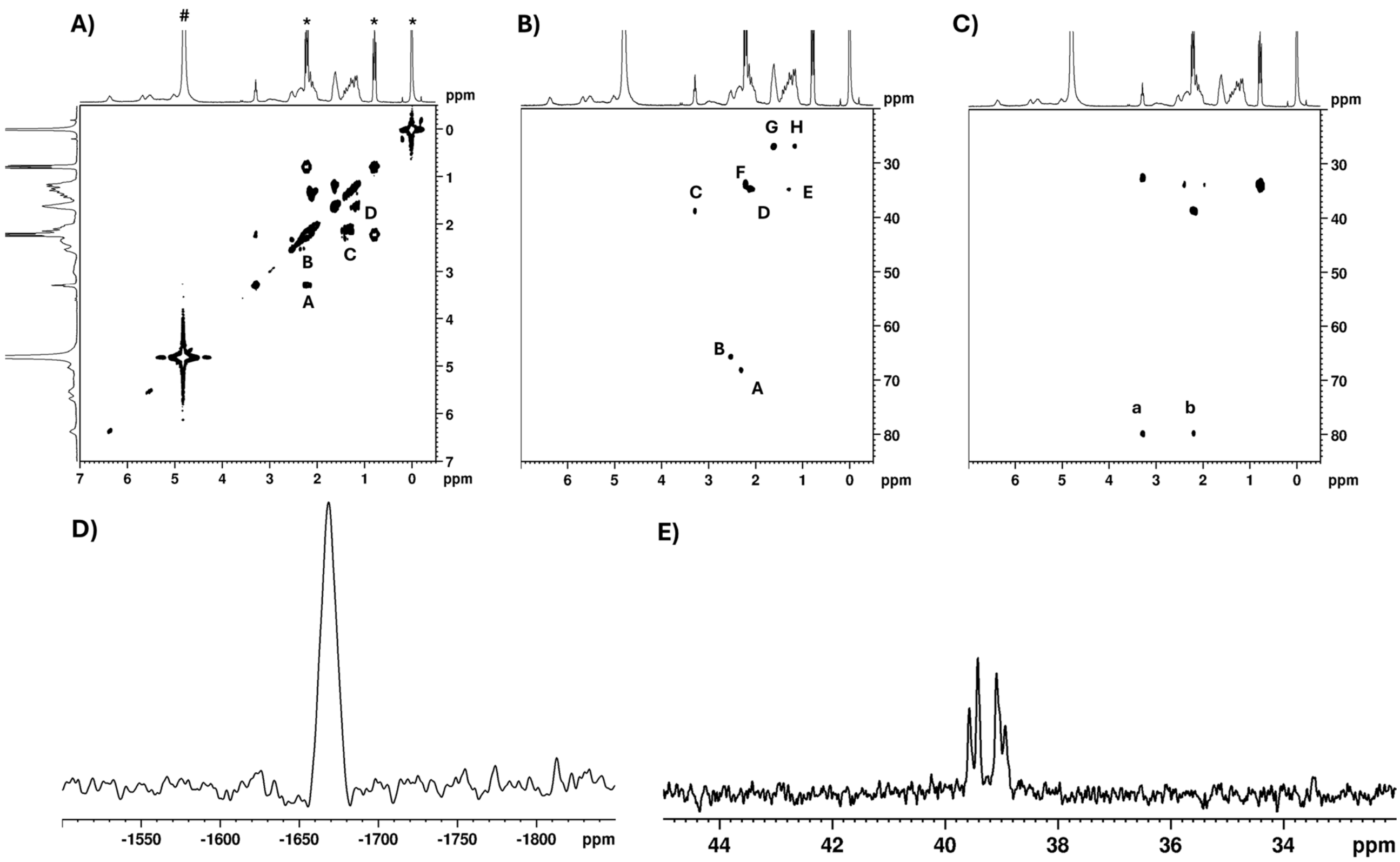

Figure S1. A) [^{1}H-^{1}H]-COSY (300 MHz), B) [^{1}H-^{13}C]-HSQC (75.5 MHz, ^{13}C), C) [^{1}H,^{13}C]-HMBC (75.5 MHz, ^{13}C), D) $^{195}Pt\{^{1}H\}$ (64.5 MHz), E) $^{31}P\{^{1}H\}$ (121.5 MHz) spectra of **PtBP3** in D_2O. The asterisks indicate the TSP signals and # indicates the residual solvent peak.

Characterization of [{Pt(cis-1,4-DACH)}$_2$(PAM)]NO$_3$ (PtBP4)

PtBP4 was characterized by elemental analysis, ESI-MS, and ^{1}H,^{31}P,^{195}Pt, [^{1}H,^{1}H]-COSY, [^{1}H,^{13}C]-HSQC, and [^{1}H,^{13}C]-HMBC NMR. The ESI-MS(+) spectrum showed the presence of a peak at *m/z* = 850.0787, corresponding to [**PtBP4**+H]$^{+}$, whose isotopic pattern is in excellent agreement with the theoretical one (data not shown). The elemental analysis is also in agreement with the presence in the complex of a pamidronate residue and two residues of [Pt(*cis*-1,4-DACH)]$^{2+}$. The [^{1}H,^{1}H]-COSY spectrum of **PtBP4** in D_2O (Figure S2A) shows a cross-peak at 4.96/5.31 ppm (A in Fig. S2A), assigned to the protons of the amino groups of coordinated *cis*-1,4-DACH. The cross-peak at 3.31/2.24 ppm (B), is assigned to the bisphosphonate H(3')/H(2') protons. Finally, the cross-peak at

3.14/1.87 ppm (C) is assigned to the protons H(1/4)/H(2/3/5/6). The ^{31}P NMR spectrum of **PtBP4** in D_2O (Fig. S2E) shows a singlet at 38.84 ppm assigned to the two phosphorus atoms of PAM. Contrary to what observed for **PtBP3**, the P atoms are magnetically equivalent due to the absence of chiral carbons in the carrier ligand *cis*-1,4-DACH and resonate at lower fields ($\Delta\delta$ = 21.02 ppm) with respect to those of free PAM. The ^{195}Pt NMR spectrum of complex **PtBP4** (Fig. S2D) shows a broad signal falling to -1596.57 ppm. Similarly to what observed for **PTBP3**, the two platinum atoms have coincident chemical shift giving rise to a single broad signal. The NMR characterization of the carbon atoms of **PtBP4** was carried out by recording a [^{1}H,^{13}C]-HSQC spectrum (Figure S2B). The cross-peak which falls at 49.65/3.14 (^{13}C/^{1}H) ppm (A in Fig. S2B) is assigned to C(1/4)/H(1/4). The cross-peaks at 38.92/3.31 (B) and 32.85/2.24 (C) (^{13}C/^{1}H) ppm are assigned, respectively, to C(3')/H(3') and C(2')/H(2'). Finally, the cross-peak at 24.78/1.87 (^{13}C/^{1}H) ppm (D) is assigned to C(2/3/5/6)/H(2/3/5/6). To conclude, in order to assign the ^{13}C chemical shift of quaternary C(1') a [^{1}H,^{13}C]-HMBC spectrum was recorded (Fig. S2C). This spectrum presents two cross-peaks falling at 78.22/3.31 (a) and 78.22/2.24 (b) (^{13}C/^{1}H) ppm, assigned to C(1')/H(3')and C(1')/H(2'), respectively.

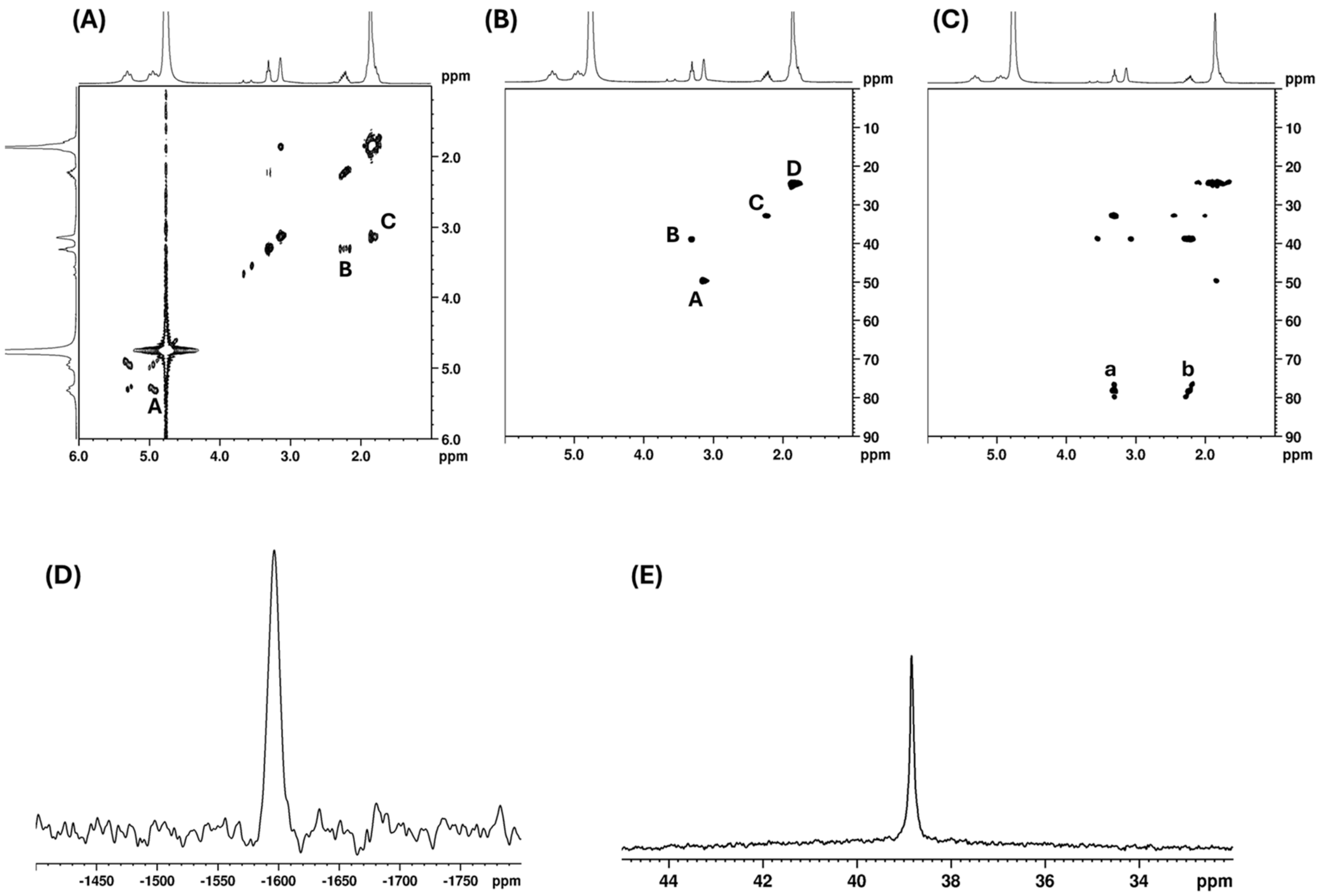

Figure S2. A) [^{1}H-^{1}H]-COSY (300 MHz), B) [^{1}H-^{13}C]-HSQC (75.5 MHz, ^{13}C), C) [^{1}H,^{13}C]-HMBC (75.5 MHz, ^{13}C), D) ^{195}Pt{^{1}H} (64.5 MHz), and E) ^{31}P{^{1}H} (121.5 MHz) spectra of **PtBP4** in D_2O.

Reaction of complex **PtBP4** with 5’-GMP (^{31}P NMR)

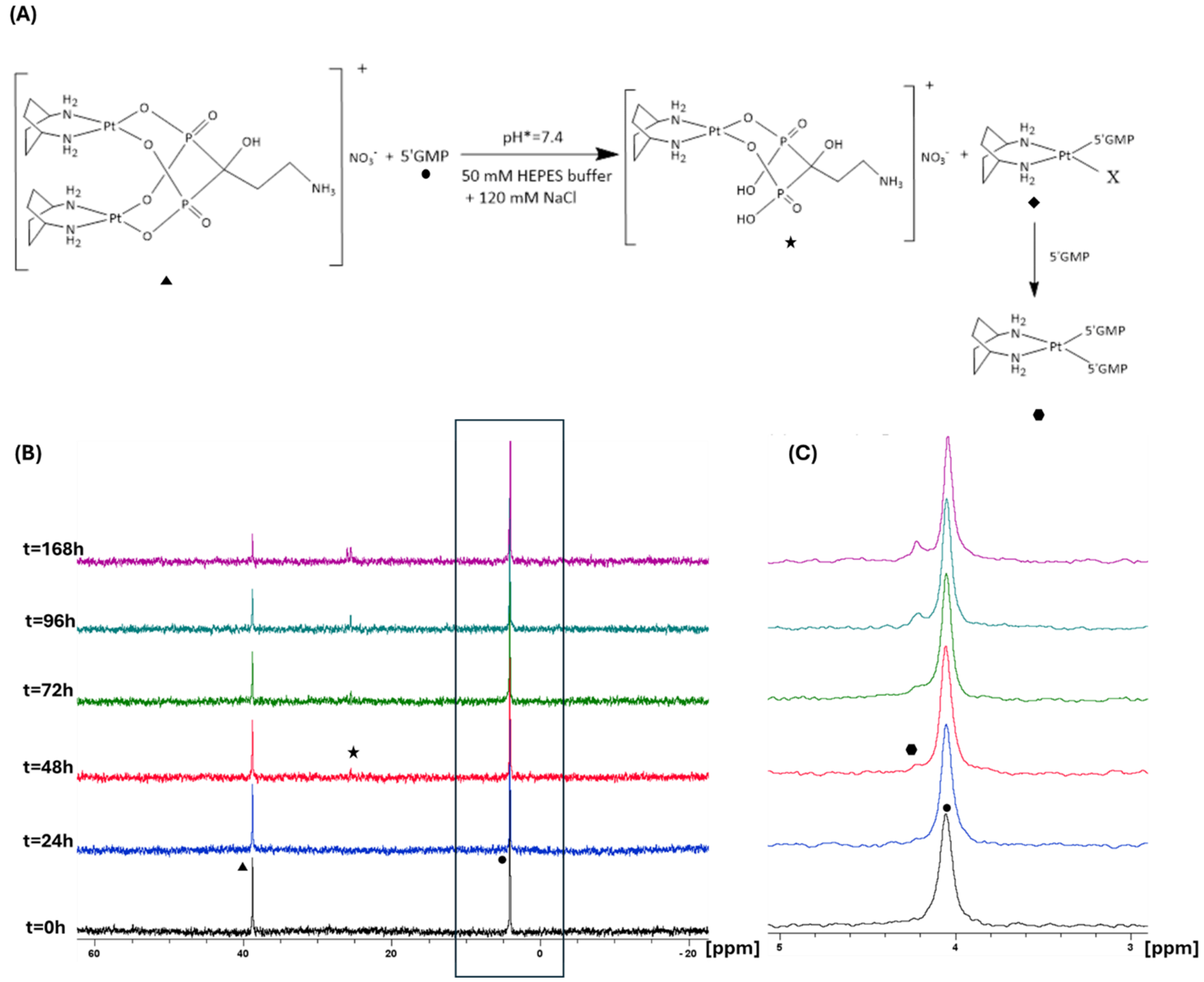

Figure S3. A) Scheme of the reaction of complex **PtBP4** with 5’GMP; **B)** ^{31}P NMR spectra of the reaction of **4** with 5’-GMP at different time intervals; **C)** Magnification of the box indicated in (B).

For **PtBP4**, the reaction with 5’-GMP was slower compared to **PtBP3**. ^{31}P NMR spectrum recorded after 48h, (Figure S3B) shows, in addition to the signal of **PtBP4** (indicated with a triangle) the appearance of a new signal at 4.33 ppm (indicated by a hexagon in Figure S3C), assigned to the phosphorus atom of the 5'-GMP of the adduct [Pt(5'-GMP)$_2$(*cis*-1,4-DACH)] and a signal at 25.48 ppm, assigned to the mononuclear species Pt-PAM (indicated with a star in Figure S3B). The intensity of these signals increases progressively over time; furthermore, it is interesting to note that the ^{31}P NMR spectrum recorded after 168h shows the improvement of the resolution of the mononuclear Pt-PAM adduct (25.99 and 25.46 ppm). Contrarily to the reaction of **PtBP3** with 5’-GMP, the recording of a ^{31}P NMR spectrum at higher fields (500 MHz,1H; spectrum not shown) did not allow to detect the monoadduct [Pt(5'-GMP)X(*cis*-1,4 -DACH)].

Reaction of complex **PtBP4** with 5'-GMP (^{1}H NMR)

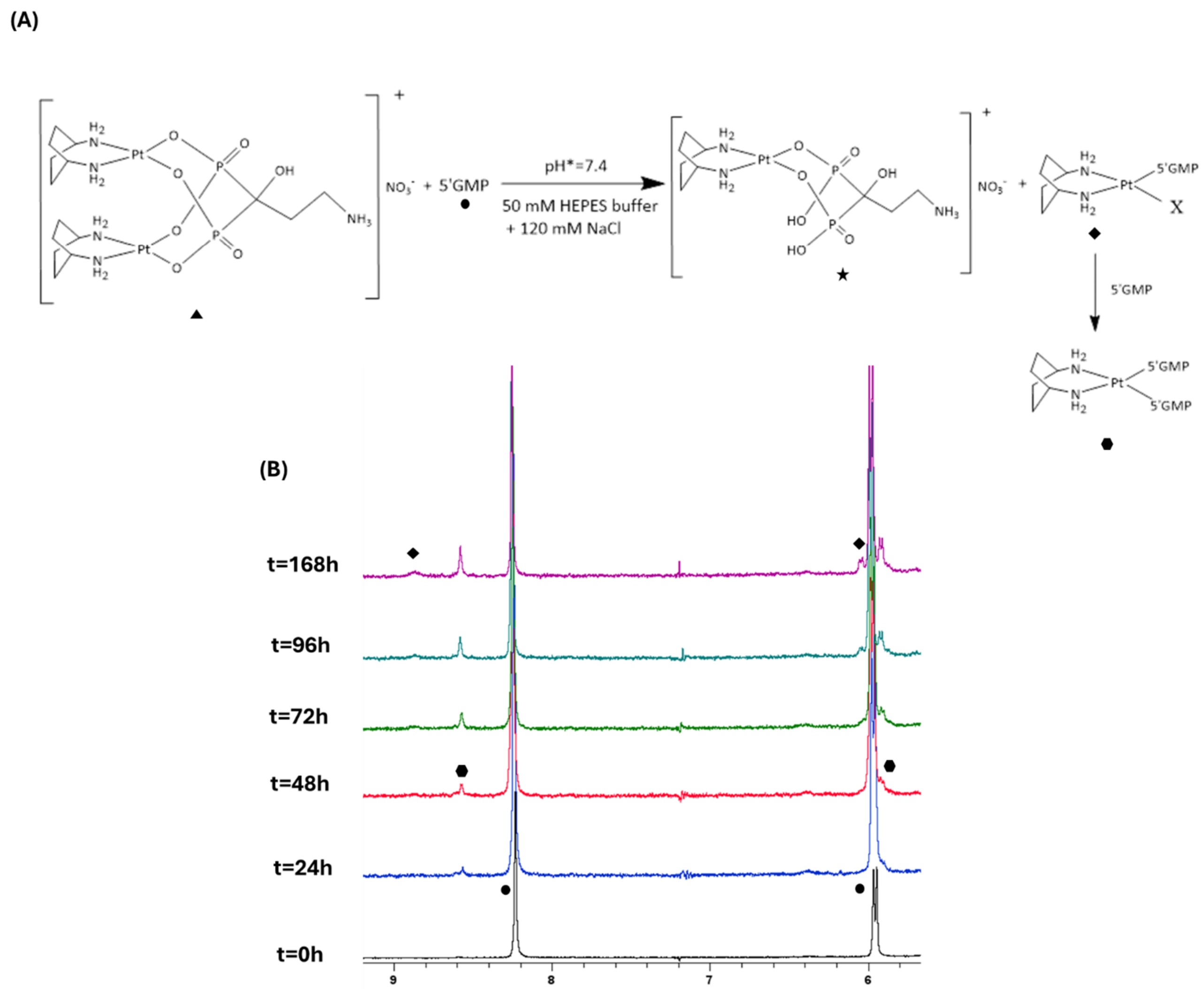

Figure S4. **A)** Scheme of the reaction of complex **PtBP4** with 5'-GMP; **B)** ^{1}H NMR spectra of the reaction of **PtBP4** with 5'-GMP at different time intervals.

In figure S4, the spectrum recorded at t = 0h shows only the signals belonging to free 5'-GMP: a singlet at 8.20 ppm and a doublet at 5.92 ppm (indicated with a circle), belonging respectively to the protons H_8 and $H_{1'}$ of guanine. After 48 h it was possible to detect the presence of two new signals (indicated with a hexagon), respectively at 8.57 and 5.91 ppm, which can be assigned to the protons H_8 and $H_{1'}$ of the two coordinated 5'-GMP residues in the bis-adduct [Pt(5'-GMP)$_2$(*cis*-1,4-DACH)]. These signals increase in intensity over time and after 168 h it is possible to detect two new signals that fall at 8.87 and 6.04 ppm (indicated with a diamond), assignable as in the case of **PtBP3**, to the protons H_8 and $H_{1'}$ of the coordinated 5'GMP in the monoadduct [Pt(5'-GMP)X(*cis*-1,4-DACH)(PAM)] (X = Cl or H_2O).

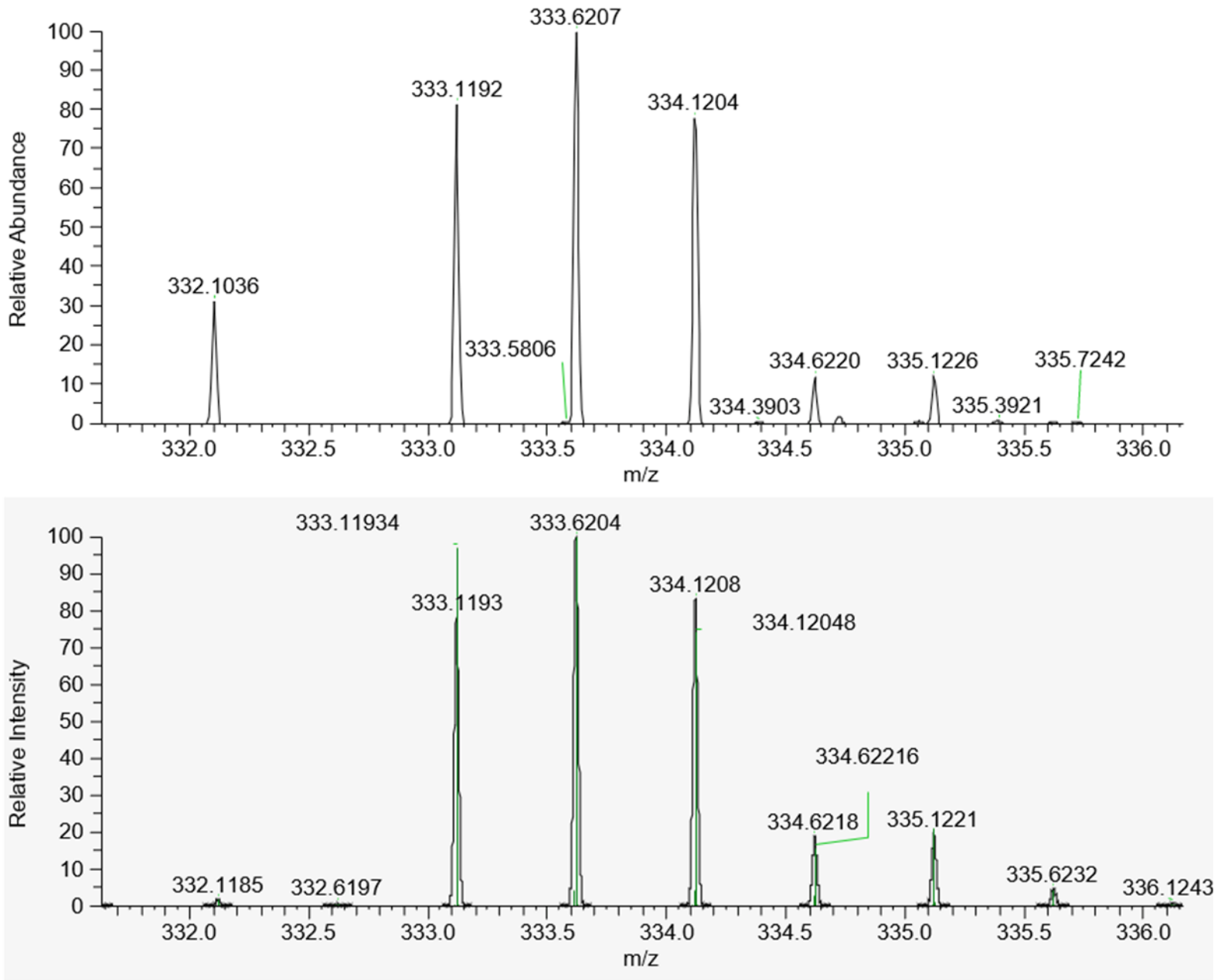

Figure S5. ESI-MS of **PtBP4** incubated with 9-EtG for 24 h at 37 °C. Adduct of **PtBP4** with two 9-EtG: found: 333.6205, calcd: 333.6205 (M for $C_{20}H_{32}N_{12}O_2Pt$, charge 2+)

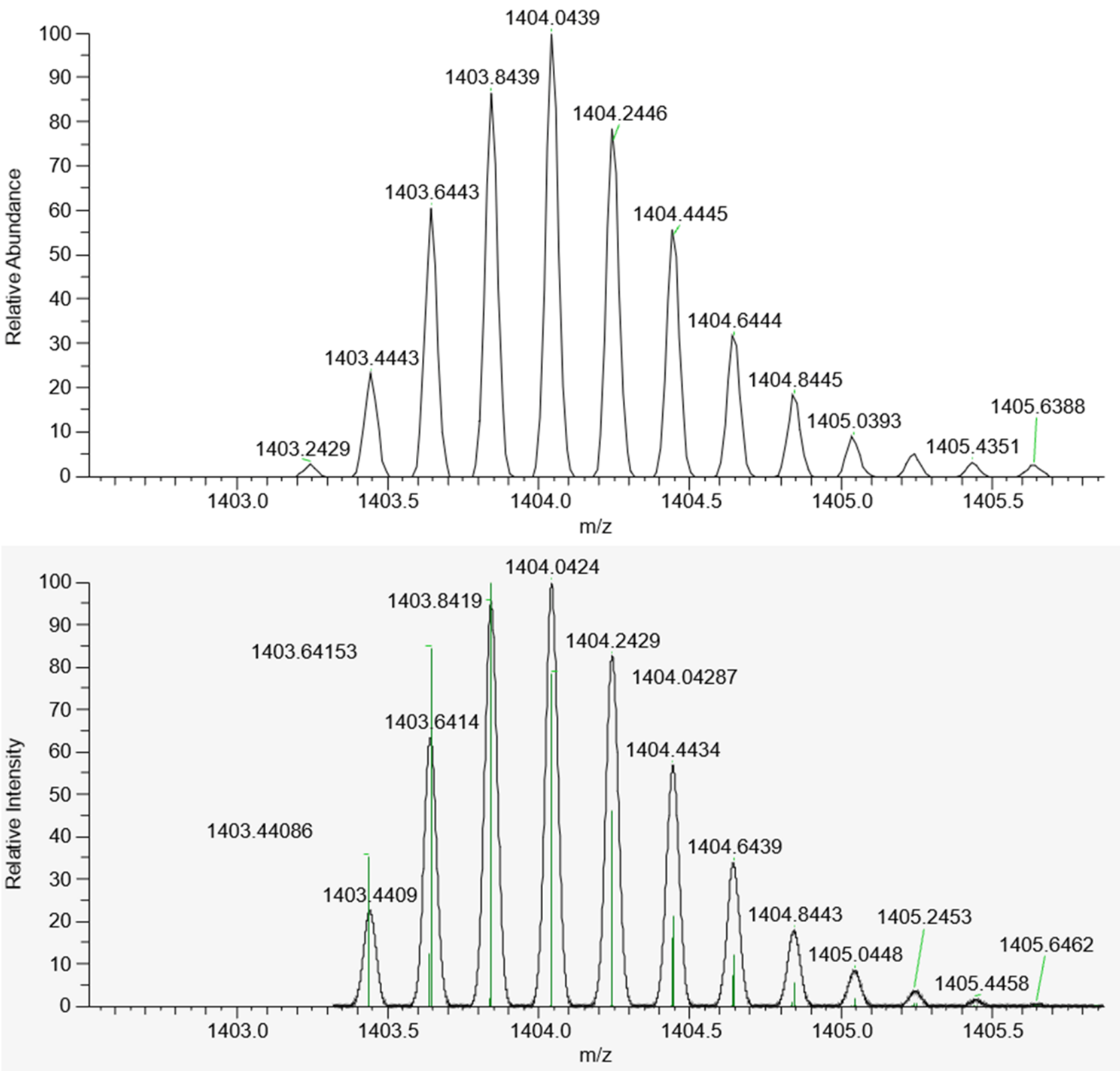

Figure S6. MS of c-Myc. Five charged masses of c-Myc with two NH_4^+: found: 1404.0439, calcd: 1404.0424 (M-H for $C_{220}H_{268}O_{131}N_{95}P_{21}(NH_4)_2$)

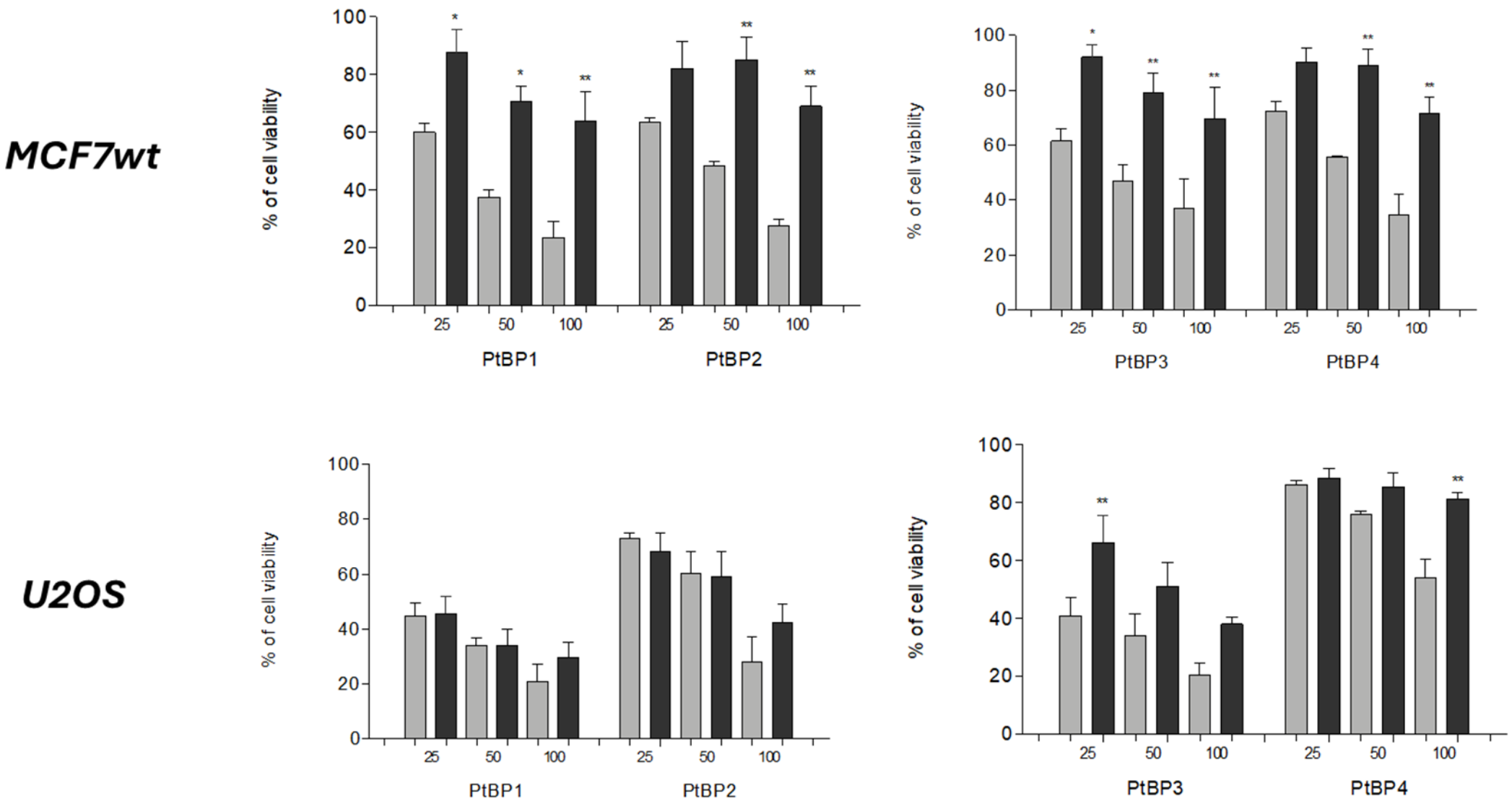

Figure S7. Effect of α-tocopherol on the cytotoxicity of PAM and ZL-containing dinuclear platinum(II) complexes in MCF7wt and U2OS cells after 72 h of incubation. Cell viability was assessed by the MTT assay, and dinuclear platinum(II) complexes tested at three different concentrations (25, 50 and 100 μM) with or without 100 μM α-tocopherol. Data are expressed as mean ± SD of three independent experiments. Statistical significance was calculated relative to treatment with the compound alone (*$p < 0.05$, **$p < 0.01$, **$p < 0.001$).
